# Wealth-relative effects in cooperation games

**DOI:** 10.1016/j.heliyon.2019.e02958

**Published:** 2019-12-03

**Authors:** Robert L. Shuler

**Affiliations:** NASA Johnson Space Center, 2101 NASA Parkway, Houston, TX, 77058, USA

**Keywords:** Prisoner's dilemma, Evolution of cooperation, Game theory, Social dilemma, Economics, Information science

## Abstract

This paper investigates cooperation games in which poor agents do not benefit from cooperation with wealthy agents. They instead benefit from considering wealth relative to decision payoffs of fitness or wealth. Of concern is the effect of cooperation on participants, their rational self-interest and choices, and not the evolution of cooperation directly. The accumulation of fitness or wealth has been shown in the literature to lead to different optimal strategies for wealthy and poor players in Chicken games. The effect could have important explanatory power if it were more broadly applicable. First we empirically compare two published results, one involving the temptation parameter vs. degree of cooperation in Prisoner's Dilemma, and the other a surprising result from a public goods game with participants from different cultures, networks and wealth in which a fixed rather than relative payoff scheme was used. Using the temptation data to calibrate the public goods behavior suggests wealth factors can provide an explanation for the results. Second we show using simulation that adding a survival threshold to a wealth or fitness accumulating Iterated Prisoner's Dilemma produces a wealth relative effect. We clarify previous results to show the poor must avoid survival risk, regardless of whether this is associated with cooperation or defection. We do this by introducing the Farmer's Game, a simulation of Iterated Prisoner's Dilemma with wealth accumulation and a survival threshold. This is used to evaluate the Tit-for-Tat strategy and four variants. Equilibrium payoffs keep the game scaled to social relevance, with a fraction of all payoffs externalized as a turn cost parameter. Findings include poor performance of Tit-for-Tat near the survival threshold, superior performance of low risk strategies for both poor and wealthy players, dependence of survival of the poor near the threshold on Tit-for-Tat forgiveness, unexpected optimization of forgiveness without encountering a social dilemma, improved performance of a diverse mix of strategies, and a more abrupt threshold of social catastrophe for the better performing mix. Lastly we compare cooperating and non-cooperating societies using the simulation and discover disturbing connections between cooperation and familiar non-egalitarian wealth distribution patterns.

## 1 Introduction

Cooperation is highly desired by many social planners, economists and political scientists. Many disciplines study how to promote it. This is a paper about its effects on individuals under survival constraints with differing resources, and subsequent effect on the social distribution of wealth. Games with a predetermined payoff matrix are frequently used to study the formation, maintenance or evolution of cooperation. The most common example is Prisoner's Dilemma, though many others are used. The games can be played on a one-shot or iterated basis, pair wise or multiplayer. Frequently the dependency of results on network relations among participants is studied. For large network and evolutionary studies a simulation approach may be used rather than actual participants, but live subjects are also recruited and their behavior studied. In this paper we will attempt to explain some puzzling behavior of live subjects, and introduce what we find into a non-evolving game to determine how it affects the wealth and health of the simulated players.

It has been found that in the general case cooperation develops under limited conditions such as kin or group selection (Hamilton, 1964; Maynard Smith, 1964; Wilson, 1974; Wilson and Wilson, 2007; Bowles and Gintis, 2011; Simon et al., 2012). Reciprocal cooperation among non-kin in excess of purely rational behavior (Nash Equilibrium) can develop in evolutionary settings without kin/group selection (Trivers, 1971; Axelrod and Hamilton, 1981). Some instinct for cooperation is evident from early emergence in infants (Tomasello and Gonzalez-Cabrera, 2017), as well as an instinct for fairness (Burns and Sommerville, 2014). In adults there is evidence of instinctive cooperation in the face of mortal danger (Rand and Epstein, 2014), and intuitive cooperation in a variety but not all cases, with also the existence of intuitive defectors, and deliberation and experience both decrease cooperation, especially for one-shot games where reciprocity is not a factor (Bear and Rand, 2016; Rand, 2016). Culture, especially its co-evolution with cooperation, plays an important role as well (Boyd and Richerson, 2009). Social learning likely plays a role in the formation of patterns on which cooperation or conflict may be based (Pulliam, 1982), though recent evidence suggests that direct social learning of cooperation strategies may guide behavior toward individual utility maximization strategies at the expense of costly cooperation (Burton-Chellew et al., 2017). For review articles of the field see Perc and Szolnoki (2010) and Perc et al. (2013).

Perc and Szolnoki (2015) report that a diversity of punishment strategies is more effective in deterring defection (which they term crime), but leads to an explosion in complexity in the system with oscillation of ordinary, punisher and crime dominated phases and still fails to ensure cooperation (eliminate crime completely). If wealth effects lead players to different mixes of cooperation and defection strategies, then such instability is likely also to depend on wealth effects.

## 2 Problem description

This paper first addresses the problem of experimental confirmation of the results of research into the analysis of cooperation using game theory in which culture, networking and wealth differences are present. We examine data from two papers, one conducting experiments with live participants, the other based on simulations using both real networks and randomized modifications of those networks. Then we test the hypothesis that the two can be reconciled by assuming a wealth effect.

Game theory frames decision problems in an exact but limited way so that concepts are clear and theorems can be proved. It is implicit that the game in question is a complete description of the options available to the players; otherwise they might elect not even to participate, but to make investments elsewhere. It is also assumed that only the relative magnitudes of payoffs relative to each other are relevant. Experiments with human subjects rarely meet these criteria.

Since defining the complete environment of test subjects as a game is usually infeasible, then comparison among simulated, experimental and in-situ data is problematic. Without such comparison, cooperation theory based on game theory can hardly make definite analyses of real world problems, except in special cases where participants either behave as if the subject game were critically important, or their existence is dependent on it and little else. That includes other factors in the environment, and any stored payoff (wealth or fitness) they may have. For these reasons game/cooperation theory has been remarkably successful in some areas while inexplicably failing in others, the failures usually attributed ad hoc to non-rational behavior.

There has been some success with evolutionary simulation approaches in predicting not strictly rational behavior. Non-kin reciprocal cooperation has been found to develop and to depend on either small group size (Maynard Smith, 1976; Killingback et al., 2006), or heterogeneous or Small-World networks (Pacheco and Santos, 2005) so that clusters of cooperators can fend off defectors (Lozano et al., 2008). Defectors, in this context, make purely rational choices. Persistent links in a large network have been found to be sufficient (Axelrod et al., 2002).

Lozano et al. (2008) studied two networks from the real world using simulation and Prisoner's Dilemma, considering both the actual network and a randomized version of each network which preserved node degree but not clustering. One was an EMAIL network with a high degree of connectivity within and between communities, and the other a PGP (pretty good privacy) network that was more hierarchical. The real EMAIL network and the randomized versions of both networks had similar relations between the temptation parameter *b*, starting with 0.95 density of cooperators in both randomized cases, to 1.0 density of cooperators in the real EMAIL network for *b=1* (no temptation, defection payoff same size as cooperation payoff). Density decreased slowly at first and then more rapidly to a range of 0.15–0.3 for *b=2* (defection payoff double the cooperation payoff).

**Figure 1:**
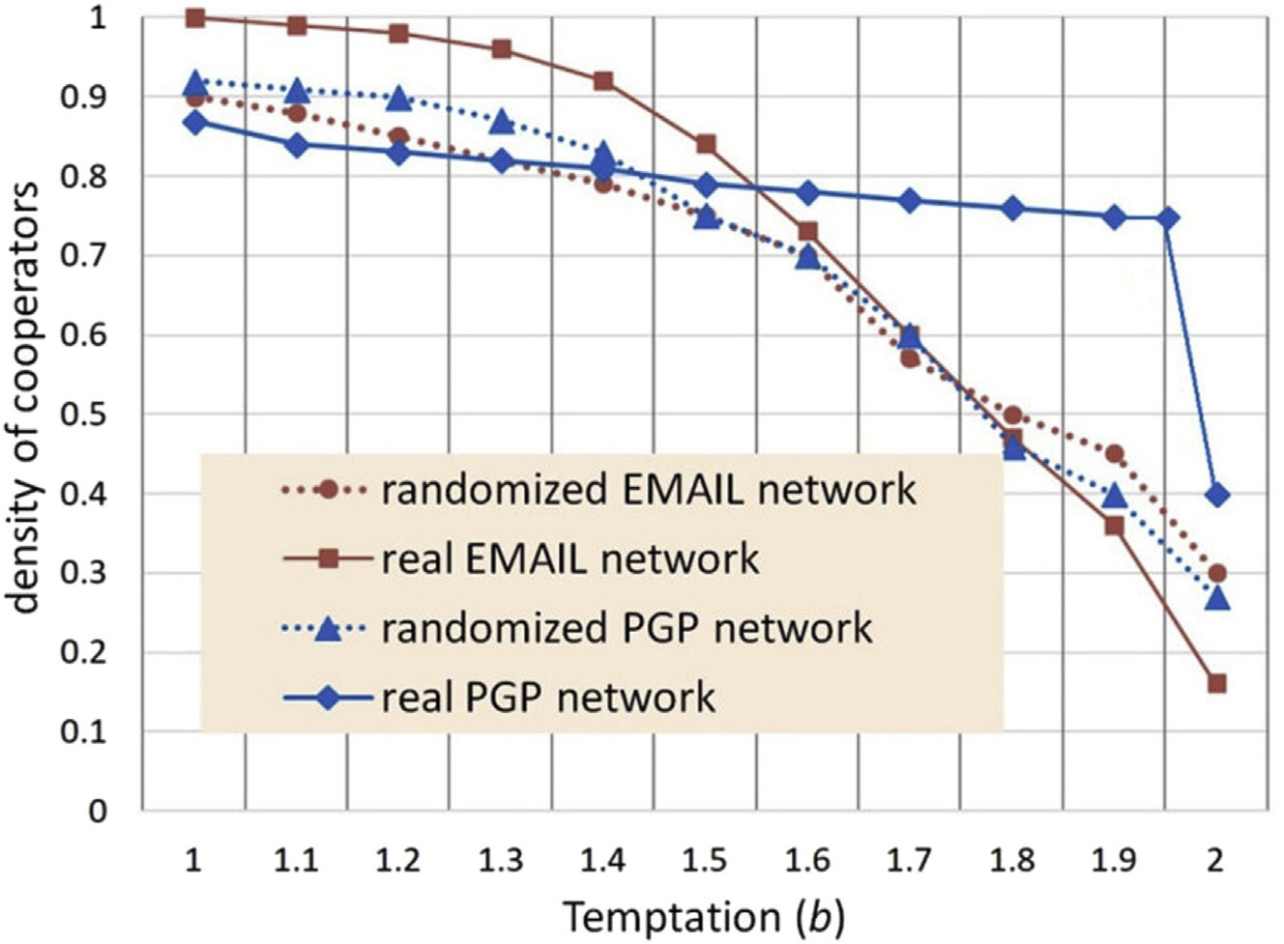
Density of cooperators derived from Lozano et al., (2008).

Figure 1 shows an enhanced comparative plot derived from separate data sets in Lozano et al. for both versions of both networks, facilitating easier understanding of the similarity in three of the cases. The real world PGP network performed differently, holding a lower level of initial cooperation relatively stable over a broad range of temptation before finally collapsing near *b=2*. The randomized PGP network was very similar to the randomized EMAIL network, illustrating Lozano's main point that global network parameters are not the most important factors in cooperation. A second point we could take from Lozano et al. may be that the relation between temptation and cooperation is similar over a broad range of networks unless they have very particular mid-scale structure (mezoscopic is Lozano's term). Other investigators such as Xu et al. (2014) report roughly similar results for cooperation as a function of temptation, even for simple lattice networks, with cooperation always vanishing below *b=2*.

Buchan et al. (2009) report on an experiment with live subjects from six different countries designed to evaluate the effects of the networks and cultural attitudes of the participants’ home countries, over a range of degrees of “globalization” which they describe as “*the increased connectivity and interdependence among people worldwide*.” The network of the experiment was a hierarchy of three LOCAL groups with four INDIVIDUALs in each, all comprising a WORLD. There were only three trials, presumably to limit social learning during the experiment, since the objective was to measure effects of the home networks, not the experimental network.

In each trial participants were given 10 tokens worth $0.50 USD each and asked to allocate them to INDIVIDUAL, LOCAL or WORLD accounts. The amount at risk over the three trials is then $15 per participant. For non-USA participants, tokens represented equivalent purchasing power in their home countries. Experimenters doubled the amounts in LOCAL accounts, dividing the proceeds among participants in the local group, and tripled the amount in the WORLD account dividing it among all participants. Thus the collective optimum would be for all participants to contribute all tokens to the WORLD account, while the Nash Equilibrium would be to invest only in the INDIVIDUAL account.

**Figure 2:**
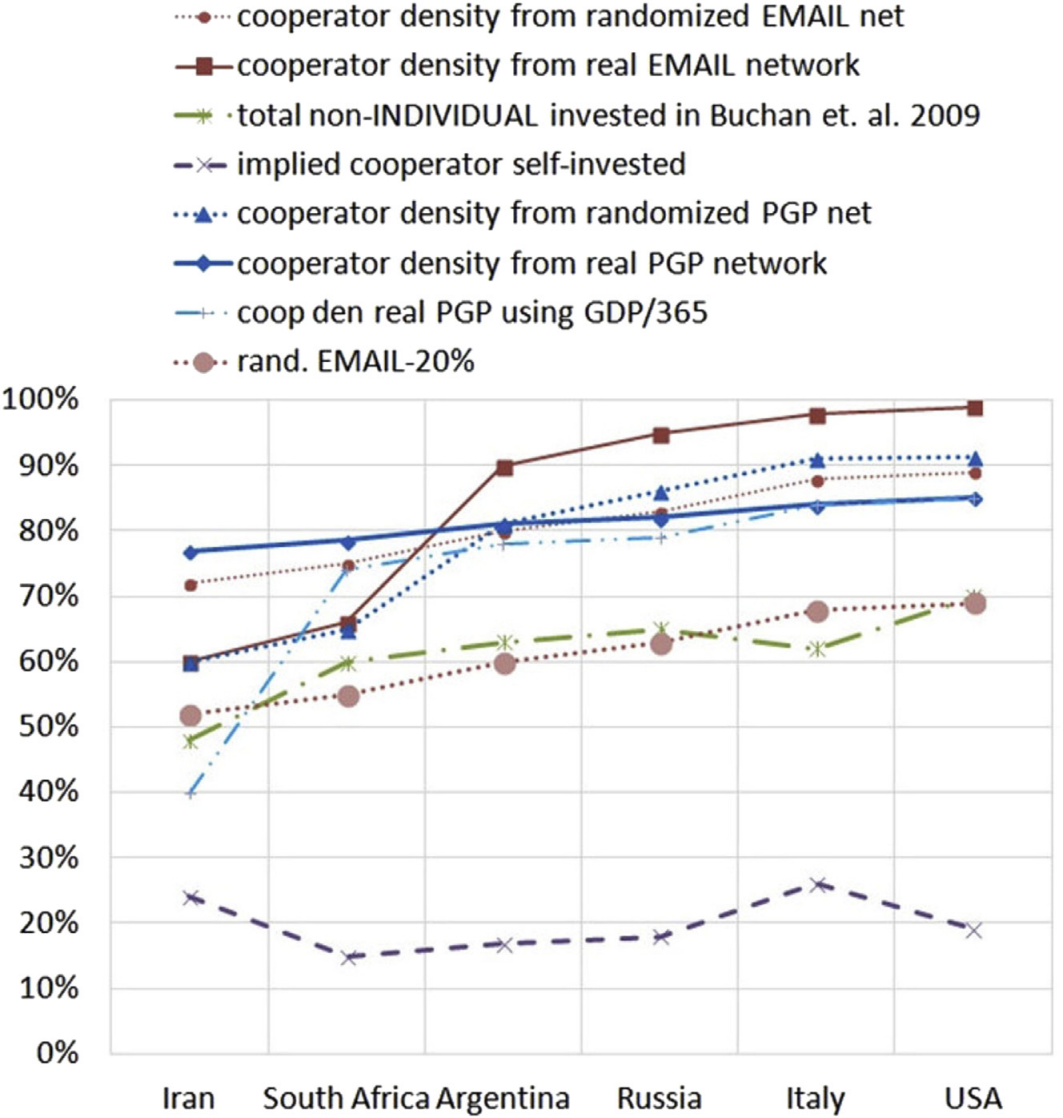
Percentage of other vs. self-investment based on analysis of Buchan et al., (2009) (investment) and Lozano et al., (2008) adjusted using GDP-relative payoff values compared to temptation.

For our purposes we sum the amount contributed to LOCAL or WORLD accounts and normalize as a percentage for an indicator of the extent of cooperation, shown as the green dot-dash line in Figure 2.

The six countries are arranged as Buchan et al. believed reflected their degree of globalization and interconnectedness, from low on the left to high on the right. The possible discrepancy that needs to be reconciled is that cooperation appears to increase as the size of connected networks increases to the right. From the simulated data on large and highly connected networks this is unexpected. If it cannot be explained, then there is a significant weakness in the simulation models, since we take the live-subject experiment to be the reference.

## 3 Reconciliation method

The problem may not only be the contextual incompleteness of the game definition. The players are not on a level playing field due to their wealth differences, which affect them personally and culturally. Ito, Katsumata, Hasegawa and Yoshimura (2017) report that in Chicken games (Hawk-Dove and snowdrift) it is *optimal* for the poor to cooperate more frequently, but not in Prisoner's Dilemma (used by Lozano et al.) or stag hunt, essentially because in Ito's analysis a wealth (fitness) parameter *w* is *accumulated*. Accumulated geometric returns are damaged too greatly by relatively greater losses (compared to existing wealth) for the poor when making risky decisions. This character is peculiar to the Chicken games. However, the principle that strategy may depend in part on a player parameter, not only on the arithmetical differences in game payoffs, means both that game payoffs are no longer independent of scale, and that strategy analysis made assuming the payoffs were independent of scale must be re-examined. Ito et al.‘s method is a well-grounded departure from elementary game theory, though it has known counterparts in economic games. For example, in a price war the player with greater stored wealth wins.

Buchan et al. use a multi-player game (twelve players in three groups) with multiple (three) and approximately continuous (ten tokens to be divided) investment choices, probably more accurately representing real cooperation problems with continuous investment choices (Killingback and Doebeli, 2002). The risk profile of this game is very different for self-investment than for other-investment (other being LOCAL or WORLD accounts, essentially a lottery depending on what eleven other participants, eight from different cultures, choose). Self-investment is a certainty while other-investment risks up to a 75% loss. This makes the Buchan et al. game like the Chicken games in a particular way. The poor should make the low-risk choice. Except in this case the low-risk choice is self-investment. If we think of other-investment as cooperation and the poor choosing cooperation, we would be misunderstanding the relatedness of the games. Their common point is risk rather than cooperation.

In Ezaki, Horita, Takezawa and Masuda (2016), who study reinforcement learning, it is found that real people (live test subjects as opposed to simulations) consider whether their payoff (in cooperation or defection) is positive or negative based on whether a priori aspirations are met, rather than its absolute value. In realistic cases, we would assume aspirations would be a proportional change to one's current wealth or fitness. This finding supports Ito et al.

In the study of crash rate as a function of economic value-risk tradeoff (Shuler, 2015a) it has been found that to explain behavior such as a factor of three difference in per capita motorway death rates between otherwise similar countries (USA and Germany), it is necessary to consider the distribution of wealth within each country and separately calculate how each segment of the population responds to a perceived tradeoff (Shuler, 2015b). This is also supportive of wealth-relative decision making, even when fatality is one of the outcomes and the decision is personal.

**Figure 3:**
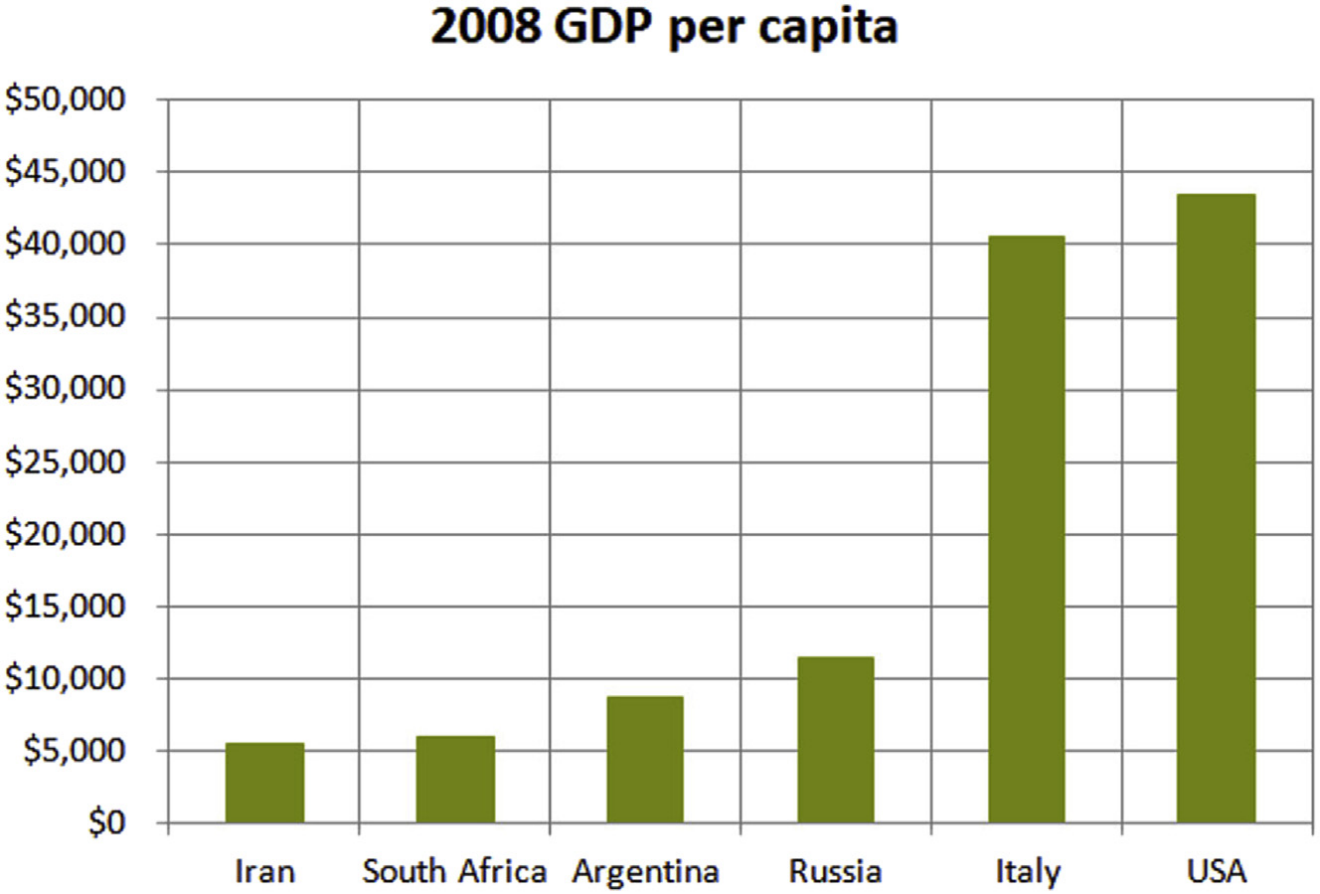
Prior year per capita GDP of countries in Buchan et al., (2009).

It seems reasonable, based on these three considerations, that we should consider the payoff values for each investment strategy in Buchan et al. relative to the wealth of the participants. We only have the country origin of the participants as a clue. This may be enough as there are large differences in the countries, and our objective is only to test whether wealth differences could explain the data, not to show they are the only explanation. Figure 3 shows the per capita Gross Domestic Product (GDP) for each participant country for 2008 (the year preceding publication of Buchan et al., in which we assume the experiment was performed, or thereabouts), which we will take as a normalization basis.

**Figure 4:**
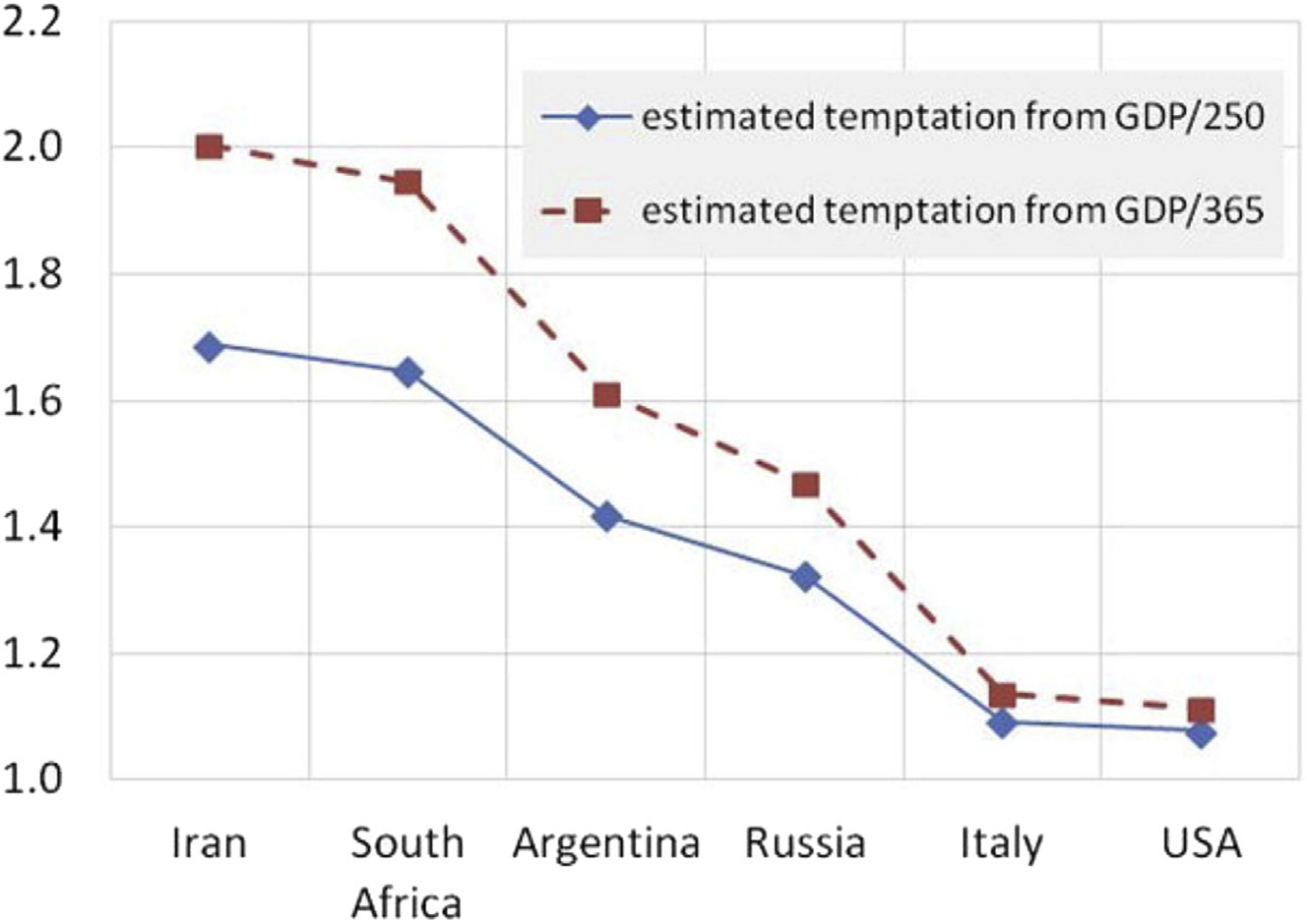
Estimated temptation as GDP/250 (workday) and GDP/125 (two workdays) normalization of $15 for each country in 2008.

There are two factors we don't know in making such a scaling. First is the threshold of what value, relative to the country, a participant considers significant enough that it should not be placed at risk even for a possibility of larger reward of a given size. In the USA $15 is an urban lunch, whereas in Iran at the time it was a day's expenses approximately. It is not the purpose of this paper to consider a methodology for accurately determining such scale factors, only to suggest that the scaling approach should be followed, so the author chose one that worked. If the country GDP is divided by the number of days in a year the significance threshold is low and temptation in the left side countries seems excessively high, close to *b=2* (see red dashed line in Figure 4).

If the country per capita GDP is divided by the number of work days, about 250, then the numbers correspond roughly to a day's wages.

The other participants were strangers and most were not visible. It has been shown that humans use facial cues even in one-shot games in choosing strategies (Eisenbruch et al., 2016), so they remained strangers.

Using the GDP/250 normalized temptation values, i.e. temptation to keep as a sure thing the $15, the temptation factors relative to average wealth in each country are found to range between 1.06 and 1.55 as shown in Figure 4. Each temptation value is used with the Lozano et al. data in Figure 1 to produce a density of cooperators estimate plotted in Figure 2. All four of Lozano's temptation curves are used with GDP/250, but only one of them with GDP/365 for comparison. We will discuss these in a moment.

The second thing we don't know is what network model to use. While the randomized network has less clustering, personal EMAIL networks may involve more discretionary choice of connections than economic networks. The more clustered real PGP network fails dramatically at high temptation. We might expect an economic network to fail less dramatically because it contains an element of necessity. One might also suppose that interaction with strangers and un-clustered networks trigger similar heuristic behavior, less cooperation in small things but declining more slowly at greater temptation.

Lastly, we ignore the difference in games and assume that participants may not even appreciate the particularities of the games, and may be mistaken at first (Burton-Chellew et al., 2015).

## 4 Results for reconciliation

Based on correlation of each candidate prediction from the Lozano simulation data weighted relative to country wealth (computed with Excel, see Table 1), the real PGP network weighted at GDP/365 has the best correlation, though all correlations are fairly good, ranging from 0.82 to 0.94.

**Table 1:** Correlation of cooperation density predictions derived from Lozano et al. temptation data GDP/250 weighted except as noted.

non-INDIV	1.00
rand. EMAIL	0.82
real EMAIL	0.84
rand. PGP	0.84
real PGP	0.85
real PGP (weighted GDP/365)	0.94

Based on visual estimation the randomized EMAIL network from Lozano et al. (2008) seems to be a reasonable predictive model for the Buchan study. In general visual estimation disagrees with the correlation data, with real PGP network weighted at GDP/365 appearing unsound for broad-spectrum prediction. There could be several reasons for this, principally the small number of country data points do not make a good statistical data set. The real PGP network has a rather violent discontinuity above temptation of 1.95, and the choice of GDP/365 positions one country just 0.05 beyond that. The direction of change between the first and second country is then correct but the magnitude far too great.

Moreover, the real PGP network is generally not a good choice not only because it may be more specialized, but because it has a sensitive dependence on calibration with respect to its near-discontinuity. Chances appear good a target experimental network will not have this discontinuity. The real EMAIL network did not, and the temptation-cooperation relations in Xu et al. did not, though those were artificial networks. The presence of discontinuities in the temptation-cooperation relations of “real” networks is an interesting question for further examination, as the presence of such discontinuities presents a problem both for forecasting and for the stability with respect to change of real networks.

Taking a linear fit approach, the overall slope of the randomized EMAIL network cooperator density function using GDP-per-capita/250 matches the slope of Buchan's actual data from live subjects, with two countries (Iran and Italy) being slightly off the linear approximation, but not so far as to be unexpected and probably due either to other cultural factors, or sampling randomness. In the author's judgment it is likely to have broader applicability, unless the target network is demonstrated to have discontinuities in its temptation-cooperation relation. The source of such discontinuities is likely to be extremely regular mid-scale structure, so that cooperation breaks down everywhere at once.

The difference between the Buchan et al. data and our Lozano et al. plus GDP derived data based on the randomized EMAIL network is shown in the dotted line at the bottom of Figure 2. This could be considered a second scale factor, a displacement constant. Whether it represents an error in predicting the density of cooperators (it might be the first time game theory models have over-predicted cooperation), or an amount withheld by the cooperators as suggested by the labeling in the figure, can be determined with additional data.

## 5 Simulation of wealth-relative effects

It is important to understand how to produce wealth-relative effects in simulation, if as our analysis suggests they are present in real data. It is also important to understand how they appear in a game such as Prisoner's Dilemma on which the temptation data was based. One clue we have already discovered is that wealth effects may be risk behavior rather than cooperation per se.

**Figure 5:**
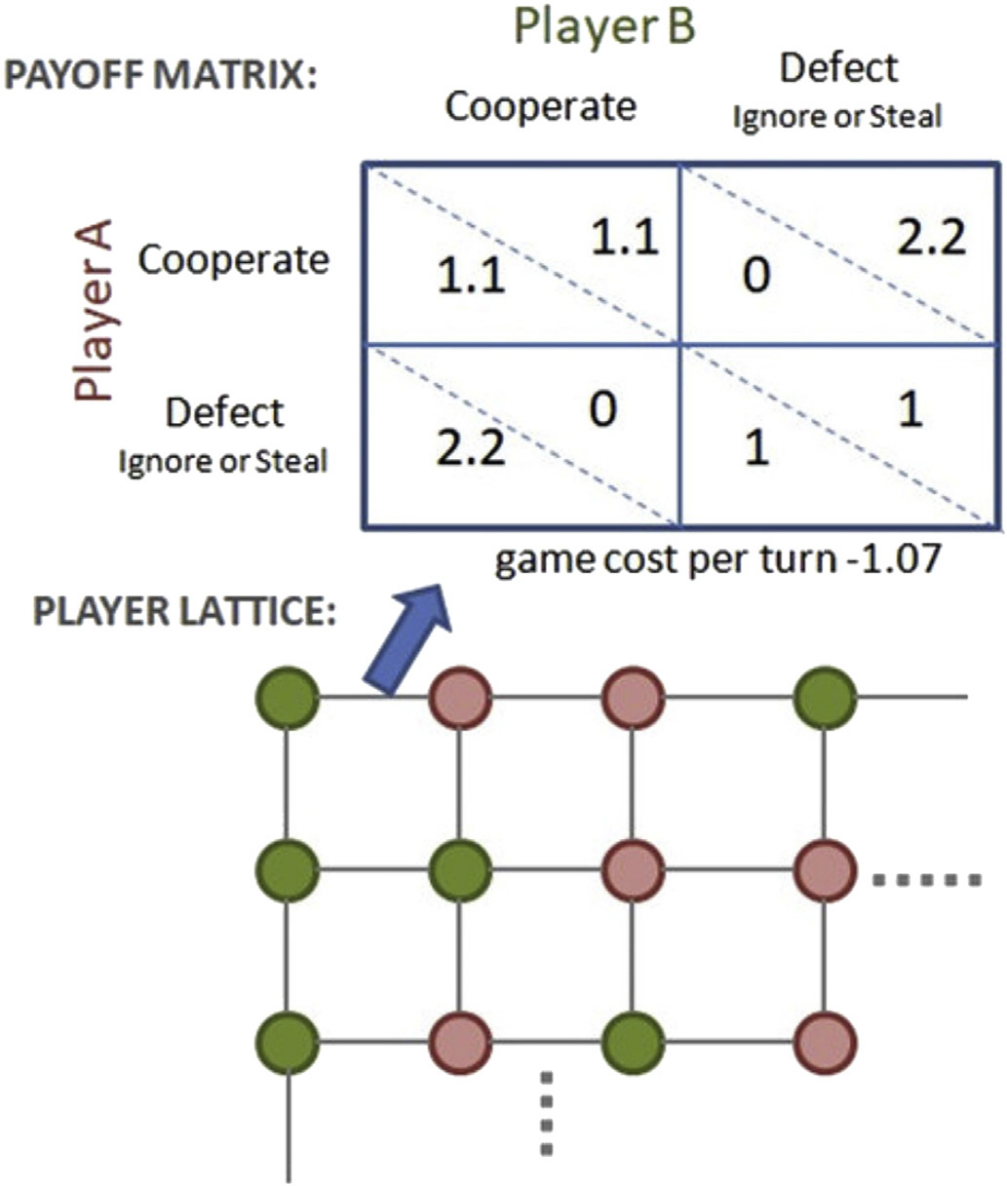
Farmer's Game payoffs and simulated network.

For these purposes a simulation was created using a tailored version of Prisoner's Dilemma which we call the Farmer's Game. It uses a payoff matrix similar to the one shown in Figure 5, and for the simulation network a lattice was used. While some investigations of the evolution of cooperation utilize dynamic networks (Rand et al., 2011) since this is a non-evolving simulation focusing on the effects of cooperation, a static network was used. Suri and Watts (2011) as well as Rand and Epstein, (2014) suggest that human cooperation does not depend excessively on network topology. The parameter “game cost-per-turn” effectively scales the entire payoff matrix for convenience (i.e.: add the game cost to each element). The simulation code is uploaded as supplementary material with this manuscript, file *simulation_code.html*, along with all data used in figures in file *simulation_data.xlsx*.

The background for Farmer's Game is that players will utilize the land between them to hunt, gather or grow food or produce other fitness-related items. If players ignore each other one token or “fitness unit” (or “food unit” if one prefers) is produced by each player as the mutual defection payoff.

If players agree to cooperate and “farm” the land, then an additional yield is available, which in Figure 5 is 10% with each player receiving a payoff of 1.1. Farming exposes both players to the risk that the other player may enter the field a few nights before the harvest and take more than his or her share. In the figure, the unilateral defection payoff of 2.2 indicates all the harvest is taken. Variations of these parameters will be considered. Note that while social dilemma games cannot be zero-sum, in most of our test cases any pair of payoffs in which at least one party is a cooperator in this game is conserved. Defection against a cooperator redistributes the benefit of cooperation, but does not remove it unless the defection is mutual. One conventional case will be demonstrated below in which the Defect-Cooperate move reduces total payoff. We might suppose the harvest is not mature or the defector loses some of it in haste.

Losing such a transaction is more than just a matter of scorekeeping due to three additional rules:

1. Players accumulate fitness (or food or wealth, etc.) proportional to their game payoffs as in Ito et al.
2. A player dropping to or below zero store of wealth or fitness tokens dies and is removed from the game and not replaced, so that we can track performance of initially assigned wealth and strategies.
3. The players must eat (or otherwise consume resources), as indicated by the game cost-per-turn, -1.07 in the figure.

It is easy to see most payoff matrices with simple integer payoffs quickly run away either negative or positive and do not remain near a death threshold, even if a game had one. So of course only the relative performance between players matters, and a run of bad choices may be turned around (though most evolutionary research simply replaces a bad strategy and continues). Evolutionary approaches may not even produce all strategies of interest.

Thus in our simulation players do not update strategies (though some strategy elements will be dependent on wealth, which is updated), and dead players are not replaced. Adjacent players are instead connected to each other for further play. There is no wraparound and being on an edge can result in slower wealth growth or decay due to fewer turns per round. All of the following results use a lattice size of 20 × 20, which has a 20% edge population. A set of Monte Carlo simulations on a lattice of 100 × 100 produced no significant changes.

Wealth is initially randomly distributed with a parameter for density of the poor. Initial poor were given 4 tokens, approximately enough to last one turn with each neighbor, and wealthy were given 10. One simulation was conducted with 8 and 20, which essentially behaves as if everyone is wealthy.

In each round (or generation) a turn is taken with each neighbor. Most effects were evident after 10 rounds and had run their course by 20 rounds. All the results below were generated using 50 rounds x 4 turns each (a “turn” being a game interaction with one neighbor, of which there are 4 except for edge players). Some results will show effects not only dependent on wealth, but on the magnitude of the cooperation excess return (10% in the figure for a 1.1 cooperation payout). This suggests another possible means of calibrating results to experimental situations based on cooperation payout. For example, in equity markets, long term mean annual return is in the neighborhood of 7.4%–12.6% (Mehra and Prescott, 2008).

Either 3 or 5 simple strategies were used depending on the simulation. These were.

1. **TitTat** – Classic tit for tat with 10% forgiveness (variable by parameter). This was implemented as a memory one strategy with each neighbor. In addition, TitTat serves as a default strategy for all the others if their special conditions are not met.
2. **Subsist** – If survival of the next round (of four turns) is seriously in doubt (present wealth 4 or less), then defect, else use TitTat strategy.
3. **Exploit** – If at least 2 times as wealthy as other player, defect, else TitTat.
4. **Thief** – If the other player is at least 2 times as wealthy then defect, else TitTat.
5. **Middle** – If survival of 2 rounds in doubt (present wealth 8 or less) then defect, else TitTat.

The primary objective is to see if and under what conditions the Subsist strategy has an advantage, as well as to generally record the history (population and wealth) of each category of players. The Exploit strategy is primarily included to create some danger in the simulation, otherwise only the poorest players would ever defect. The Middle strategy is a more conservative version of Subsist, intended in some way to represent the middle classes, though this is not verified. While the Middle and Subsist strategies are more risk averse than TitTat, the Thief strategy is more risk aggressive, attacking wealthier players.

**Figure 6:**
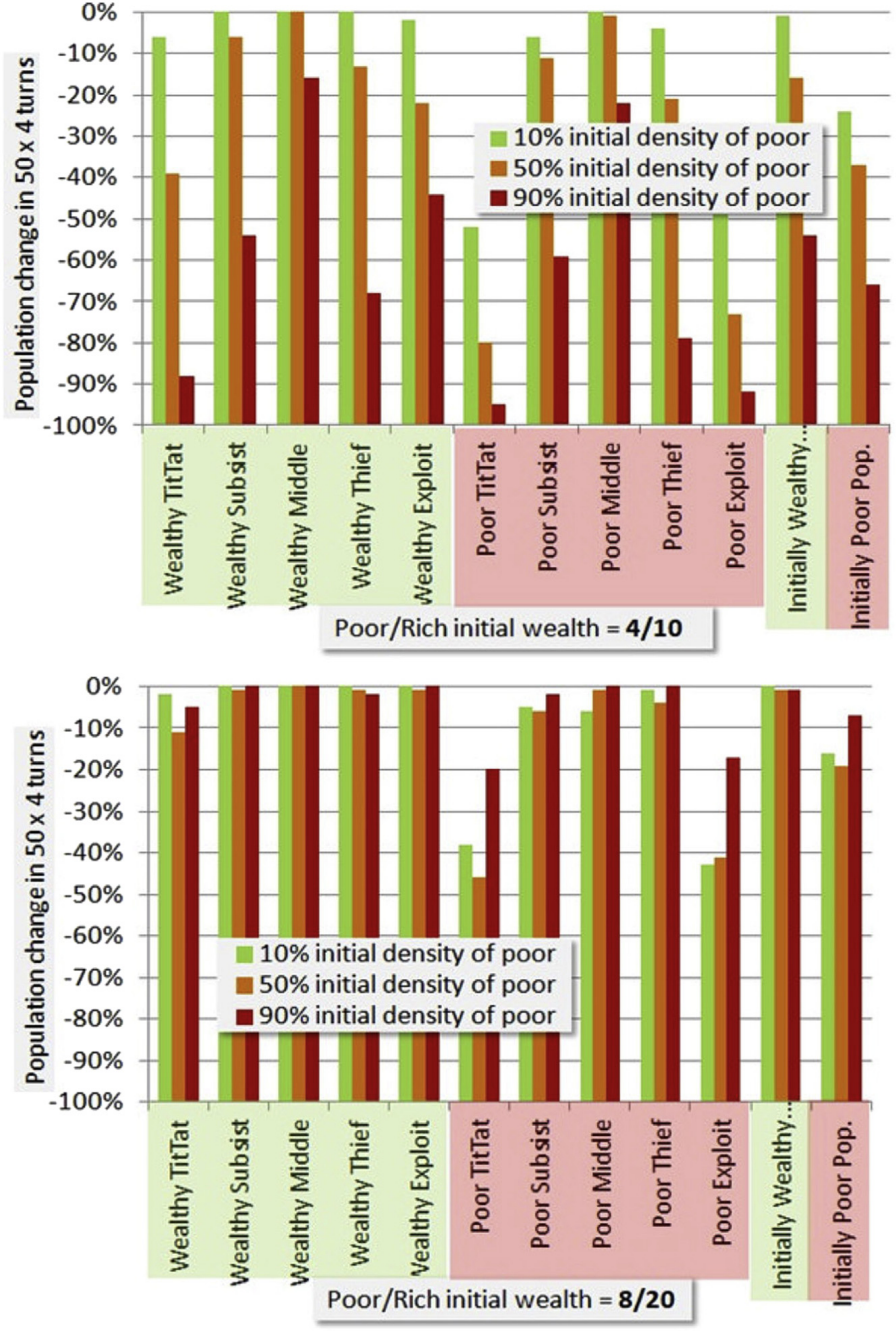
Simulated effect of variation in density of initially poor and wealthy (see legends), with cost-per-turn of 1.07 (near wealth equilibrium). Fifty iterations are employed, each consisting of a turn with each of 4 neighbors. Top figure uses the baseline of poor = 4 and rich = 10 common to other simulations to follow. Bottom figure uses double that for comparison with a case farther from the survival threshold. Relative population decline due to survival failures is shown for each strategy type, for initially wealthy and initially poor.

An overview of simulation results with respect to variation of density of the poor and magnitude of initial wealth, otherwise at the default parameters of Figure 5, is given in Figure 6 below. We will discuss those default parameters in connection with equilibrium.

There are several interesting points in this result. The initial poor/rich values of 4/10 are in a good range to show wealth effects as expected. The poor do somewhat worse than the wealthy, and the wealthy do worse if the initial density of poor is high. TitTat, is evidently the worst strategy for the wealthy in this mix. At 90% initial poor distribution, only users of the most conservative strategy, Middle, are surviving. In the 8/20 poor/rich mix, all the wealthy categories do fairly well, though again TitTat not as well as the others. For the poor, TitTat and Exploit are wiped out except for the 90% case in which the poor are mostly playing against other poor.

Keep in mind when viewing these results that they are classified with respect to initial wealth. While some players die off, or become poor, a number of players become wealthy in most simulations. However, identifying results based on final status obscures the history we are looking for. All results are based on 100 simulations at the given parameters with rich/poor status and strategy distributed randomly in each, and results averaged, using a 20 × 20 network lattice. Below we will give histograms.

**Figure 7:**
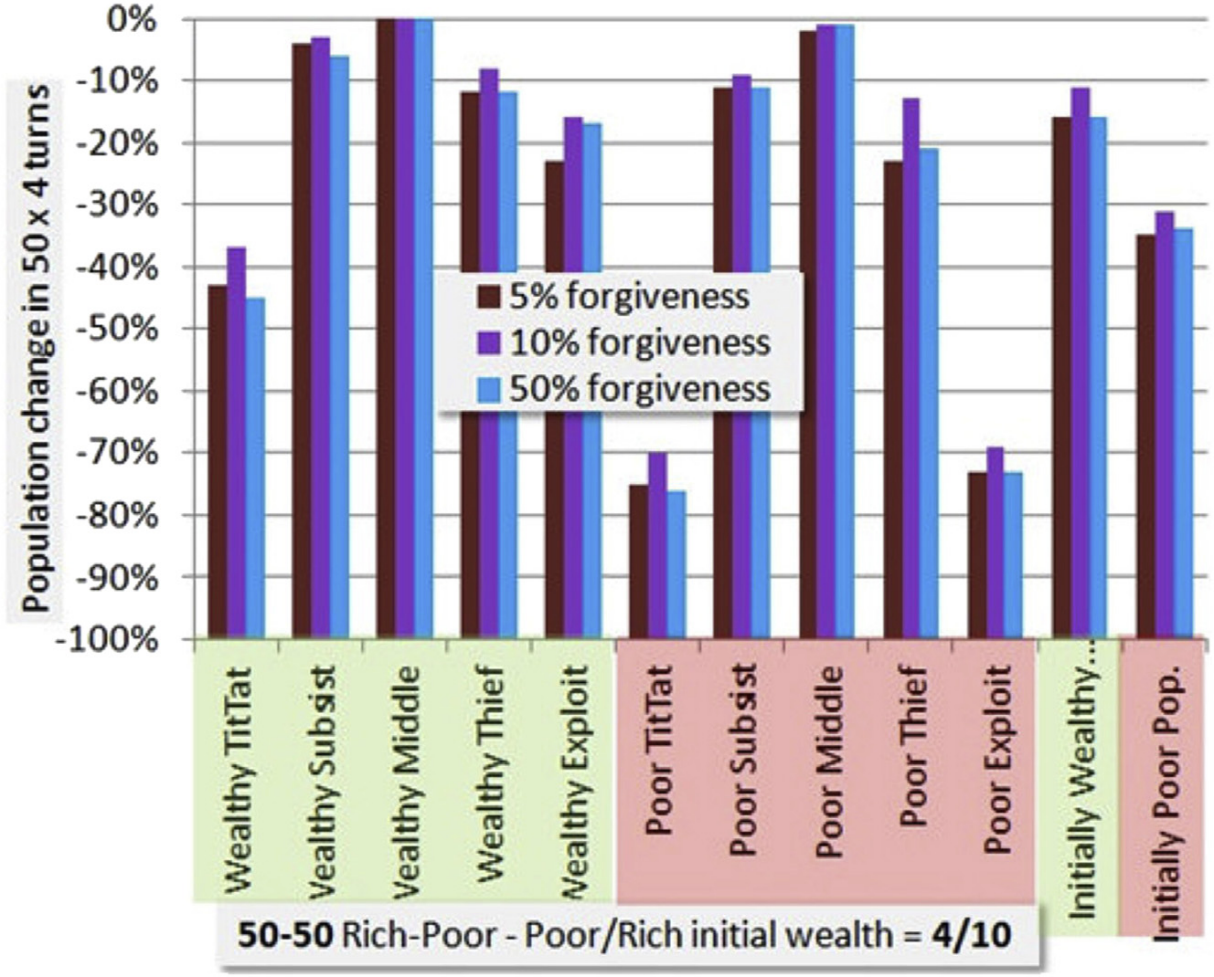
Simulation sensitivity to TitTat forgiveness. Chart is similar to Figure 6 but shows only a 50-50 distribution with baseline poor/rich values of 4/10. The three colored bars for each strategy show results for different values of forgiveness in tit for tat. Thus three separate simulations of 50 iterations are represented.

Figure 7 shows sensitivity to TitTat forgiveness. This affects all strategies, since all use TitTat is their base. The 10% forgiveness rate we have selected as our baseline gives better performance than 5% or 50%. While it is better for Thief and Exploit and Subsist as might be expected, more surprising is that it is better for everyone including TitTat, being the only condition under which any pure TitTat strategies of any wealth survive 50 rounds (~200 turns).

It appears that optimizing forgiveness, for this strategy mix, is not a social dilemma. A value which approximately optimizes the fate of a decision maker also benefits all others in terms of survival rate. Presumably wealth is less concentrated, as the forgiveness effectively shares wealth with Subsist, Middle, Thief and Exploit on occasion, so if survival is not the dominant motive there may still be a social dilemma. There is no moral justification for Exploit, since it is theft by the rich. Subsist and Thief are means for the poor to get by, to escape the apparent extinction of poor TitTat players.

**Figure 8:**
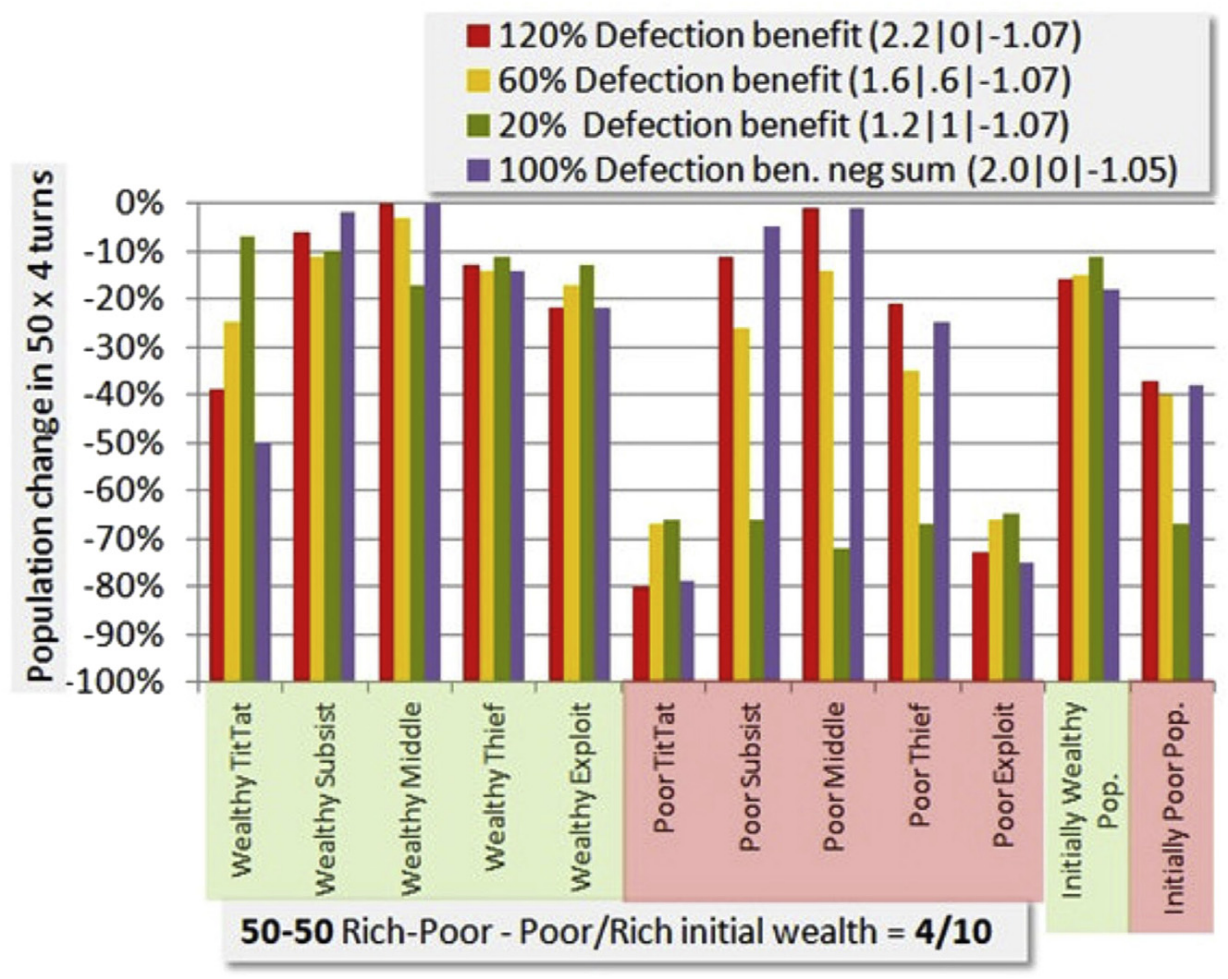
Simulation sensitivity to Defection payoff when other player Cooperates. Like the previous figure, this shows the result of several simulations varying a particular parameter, in this case the benefit to defection. The red case is the standard defection benefit used in most other simulations, and the others are lower benefits. The first two numbers in parentheses give the payoff for the defector and the victim, while the last is the turn cost used to approximate equilibrium. In the final case some of the benefit is wasted.

In Figure 8 we explore softening the impact of the Defect-Cooperate pair of moves. In addition the fourth payoff combination is the promised conventional Prisoner's Dilemma payoff in which some social benefit is lost by the Defect-Cooperate pair. In this case (2.0|0|-1.05) the equilibrium for this payoff of -1.05 cost-per-turn is used. It shows a performance similar to (2.2|0|-1.07), but just slightly worse.

In principle there is no motive for the Defector to take less than all of the harvest, as a TitTat player (which all of them are in base mode) will note one defection and present the same magnitude of response in any case. However in real life other more severe penalties may deter full defection, so we compare the baseline 2.2|0 payoff combination for D|C to 1.6|0.6 and 1.2|1. Players in this simulation were not “aware” of the payoff magnitude being changed, so this does not measure temptation to defect, which is fixed in the heuristic strategies, but changes the wealth distribution and therefore survival consequences.

There are two very striking consequences. The TitTat strategy is “rescued” by the lower impact of a partner defecting. Unfortunately this devastates the poor who depend upon the defections for survival.

If we take a straightforward notion of how the Farmer's Game payoffs are related to the temptation parameter *b*, we have Table 2 below.

**Table 2:** Temptations for Figure 8 payoffs *w=1* for Wealthy, *w=.4* for poor.

Payoffs:		Temptation:	Category:	Cooperation:
C|C	D|C	*b*=D|C/(C|C·w)	Wealthy/Poor	TitTat Survival%
1.1	1.2	1.09	W	93%
1.1	1.6	1.45	W	74%
1.1	2.2	2	W	60%
1.1	1.2	2.7	P	20%

The parameter *w* is simply the initial wealth distribution divided by 10, a weighting factor similar to what we used in connection with the empirical data. W and P are Wealthy or Poor. The negative sum payoffs are omitted because they are not consistent in design with the conservative payoffs. The data then look like a temptation vs. cooperation curve, with cooperation falling steeply just above *b=2*. This fall is due only to change in the frequency of the full cooperators (TitTat) due to die-off, since our simulation does not create new cooperators or evolve strategies, and so it is not surprising the relation is weaker than Lozano's or Xu's.

These findings already justify our hypothesis that with a death threshold and realistic cooperation benefit (not growing or declining in wealth rapidly away from the threshold) that wealth effects are evident in a game payoff matrix that qualifies as a version of Prisoner's Dilemma. But the move choice that is favored is the one which is low risk, defection, not necessarily cooperation.

**Figure 9:**
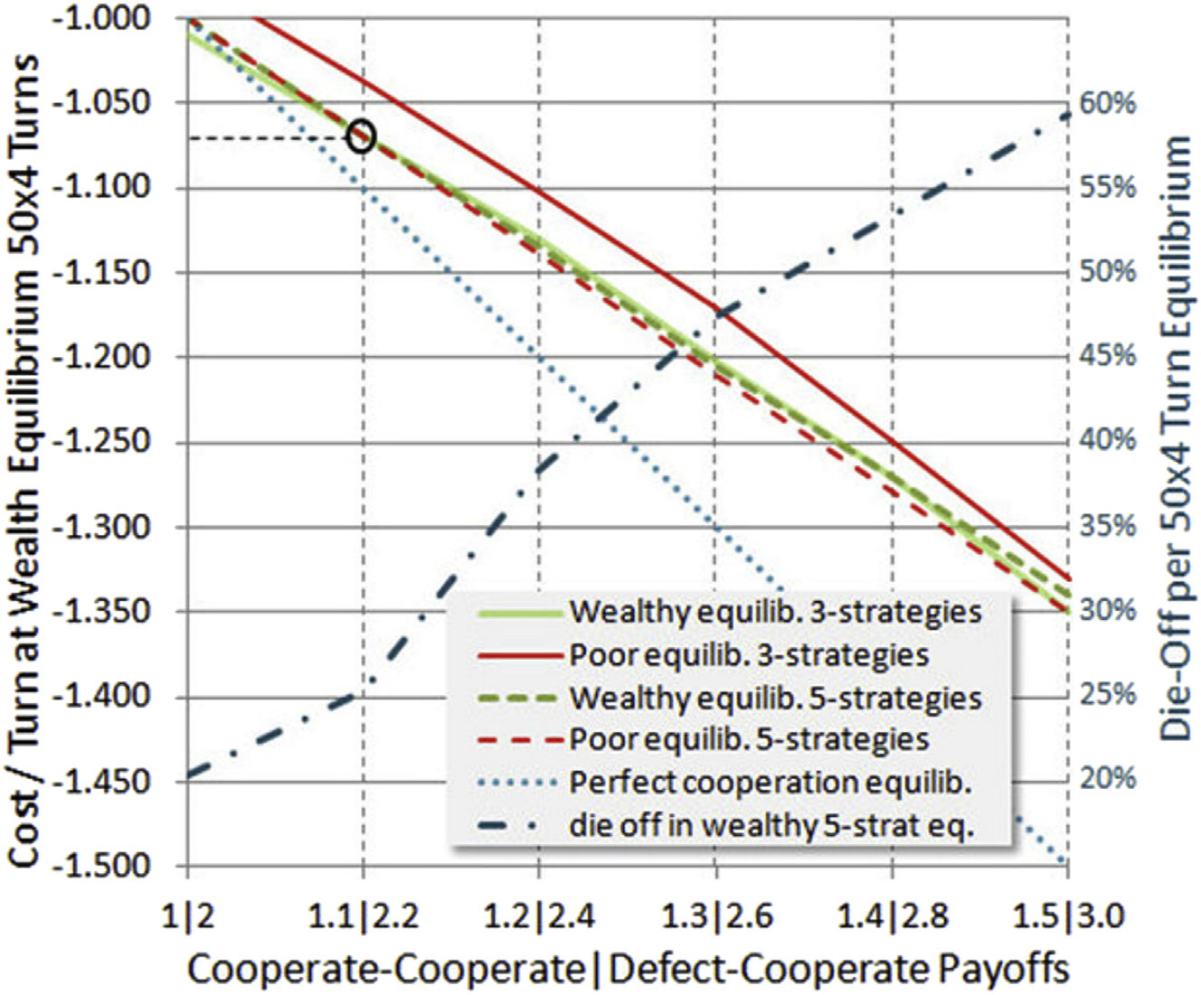
Wealth equilibrium cost-per-turn. For various game payoffs along the bottom axis, a curve is plotted which maps them to equilibrium cost per turn on the left axis (these are the down sloping lines). In addition, a mapping is provided for one case to the degree of population die off due to survival threshold. These all use the 4/10 wealth split and 50-50 wealthy-poor initial distribution. The adjective “poor” or “wealthy” in the legend indicates that equilibrium was adjusted specifically for that class. 5-strategy and 3-strategy mixes are compared (see text). In the 3-strategy case setting equilibrium for the poor produces clearly less die off, but not so much in the 5-strategy case where more sophisticated means of handling the survival threshold are available.

We now find the wealth equilibrium cost-per-turn using the 4/10 wealth split and 50-50 wealthy-poor distribution. The three strategies TitTat, Subsist and Exploit are evaluated separately from the 5-strategy mix, with results shown in Figure 9.

This analysis finds total wealth equilibrium separately for initially rich and initially poor players, for the two strategy groupings. Perfect cooperation is represented by the dotted blue line, as if all players were TitTat with no noise. In this case no player dies, however the equilibrium turn cost matches the entire benefit from cooperation.

Societies that we experience tend to exist somewhere close to equilibrium, changing slowly over time unless there is some external trigger. This means that by finding equilibrium we have a clue to an operating point. The reasons are many. Costs or conflicts might rise as populations increase or resources become scarce. Even development of new technology is a cost, one with which many are now engaged in demanding jobs. One may also view the turn cost as the standard of living, in which case a higher turn cost is not necessarily negative if the members of society can afford it.

It appears that 1.07 is the equilibrium cost-per-turn for three of the four categories, with only the poor in the less diversified 3-strategy mix having a slightly lower equilibrium. In that sense, the data of Figure 6 put the 4/10 poor at a disadvantage, since Figure 6 was based on the 1.07 equilibrium. However one can interpret that as just another wealth-relative effect: that the poor cannot keep up in a society that operates near an equilibrium standard of living, expecting certain housing and transportation and healthcare and occupational safety and so forth standards of everyone.

On the right side of Figure 9 is a scale for reading the die-off rate of one category. Die-off rates are similar for categories that have a similar equilibrium value. Perhaps the most disturbing thing about higher cooperation payoffs (benefits) is that they entail highly elevated die-off levels (or bankruptcy levels, if one is considering this to be only a financial game), unless one assumes perfect cooperation. In the author's view perfect cooperation is desirable but unlikely, and we should grapple with the reality. Evolutionary studies are needed to ascertain the likelihood, or not, of obtaining perfect cooperation, which we do not address here. We only evaluate mixes of given strategies, and specifically avoid highly tuned or optimized versions of them for generality.

**Figure 10:**
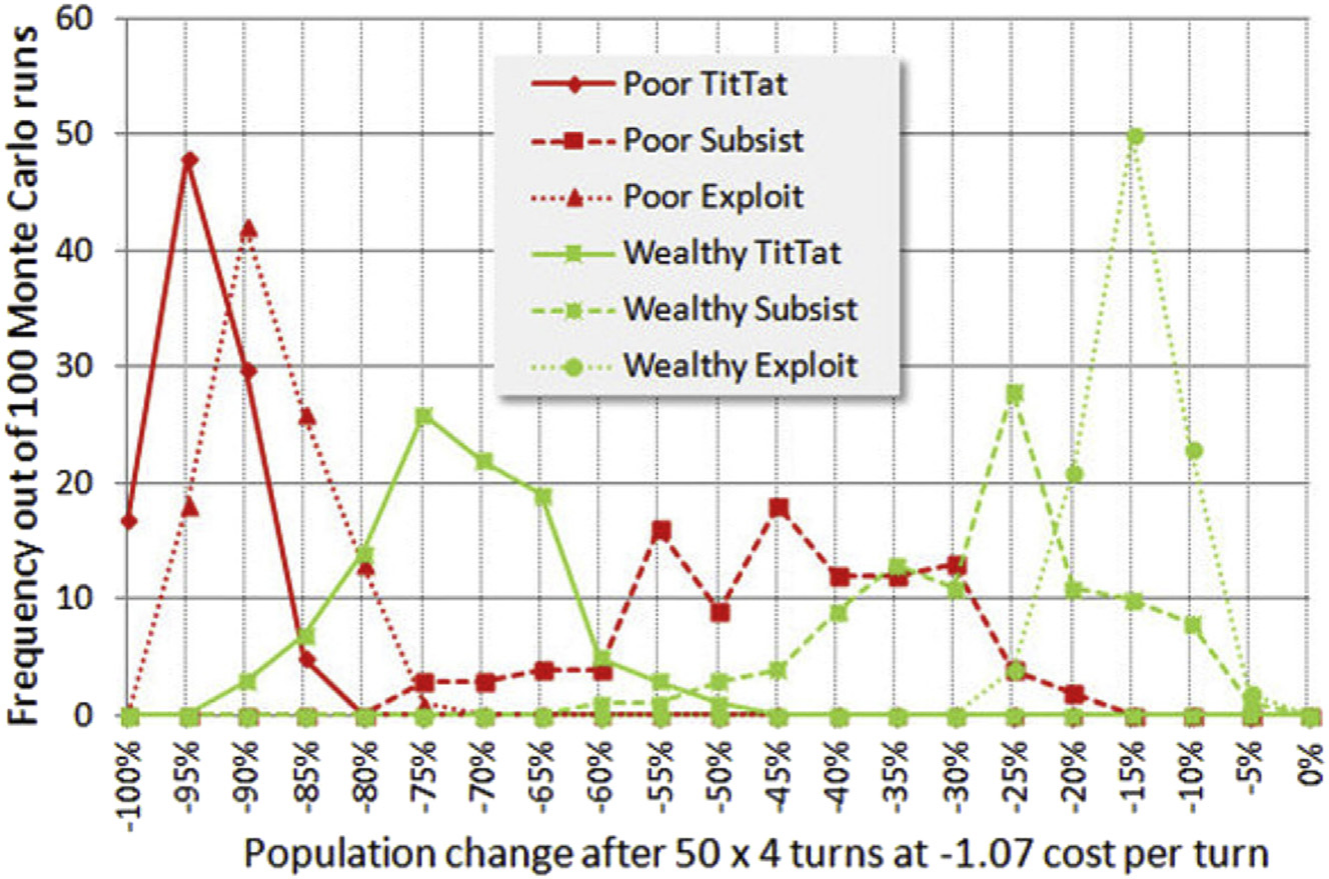
Histograms for 3-strategy mix showing final wealth distribution after 50 iterations.

Network architectures and degree of persistence in interaction are known to have large effects on strategy evolution. Without evolution we do not expect great deviation for other networks. Though persistence is high in our model, the Monte Carlo runs average a tremendous variety of configurations, such as groupings of cooperators, or lone cooperators surrounded by Subsist or Thief players. A histogram of the 3-strategy survival rates is shown in Figure 10.

Four of the histograms are quite close to normal distributions. The two Subsist categories have a high variance and are only loosely normal. They become normal at 300 or more Monte Carlo iterations, but their average value appears usable at 100 iterations, facilitating the examination of a large number of cases.

**Figure 11:**
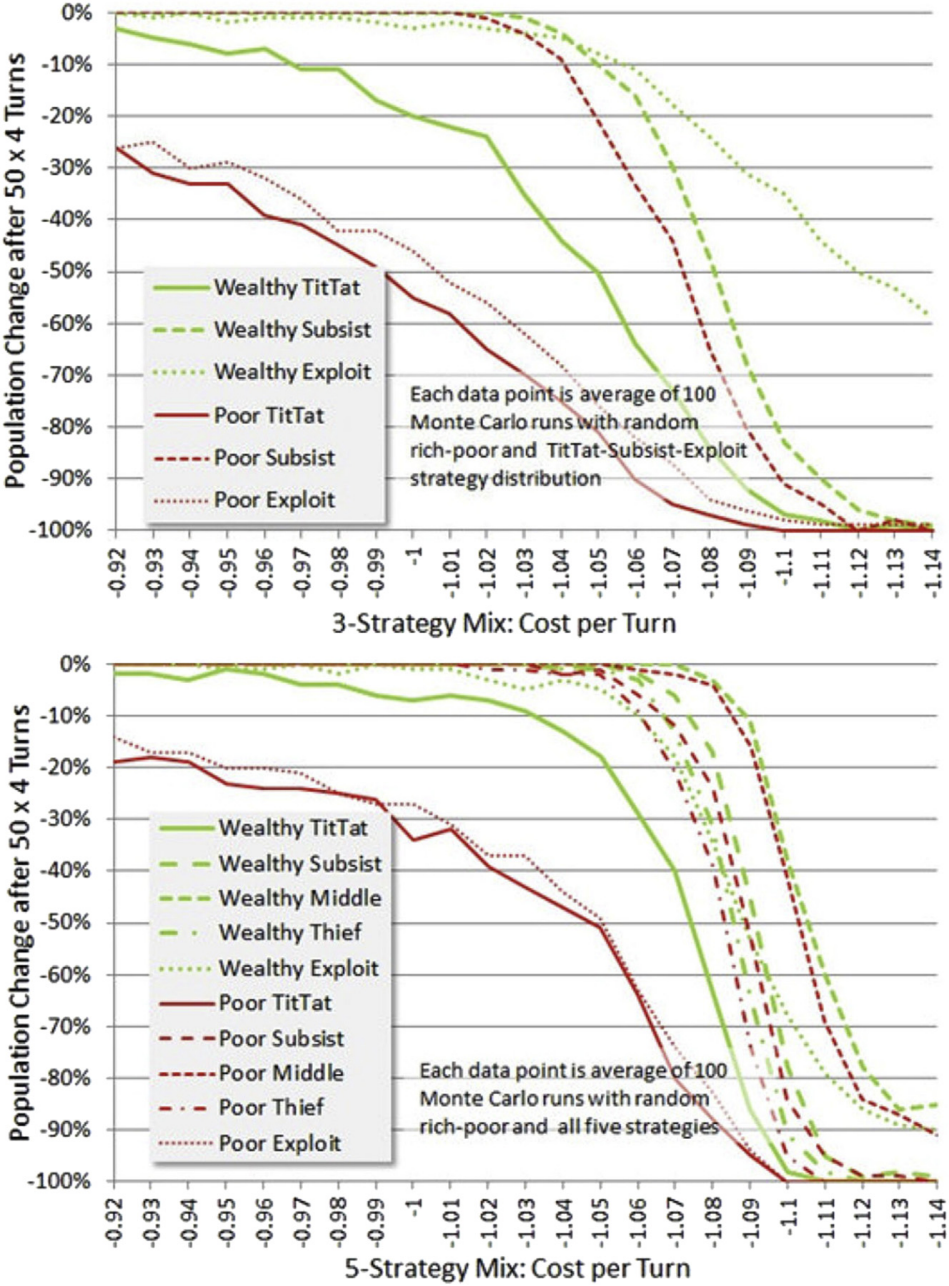
Survival vs. Cost-per-Turn at 1.1 cooperation payoff. The top figure shows final population in each of 3 strategy categories for wealthy and poor as a function of cost per turn. One can see that poor tit-tat and poor exploit strategies are doing extremely badly, while poor subsist does better than some wealth strategies. This trend continues in the 5 strategy mix below, but poor subsist performs 2^nd^ to poor middle, which does almost as well as the best wealthy strategy, also a “middle” strategy. See text for strategy explanation.

Figure 11 shows a different way of visualizing the variation of simulation performance, showing survival rates for each category vs. cost-per-turn for the baseline 1.1|2.2 mutual-cooperation | defection-if-cooperating payoff. The 3-strategy mix has evidently a different character, performing somewhat less well than the 5-strategy mix, but having a more forgiving slope as cost-per-turn increases.

The equilibrium of the poor in the 5-strategy mix improves because the added Middle and Thief strategies have substantial regions of non-diminishing population. We might tentatively ascribe a benefit to diversity of strategies since not only is the overall performance better, which might be due to the added strategies, but also strategies such as Poor-Subsist which occur in both groups perform better in the 5-strategy mix. Perhaps this is due to lower likelihood of unfavorable clusters forming, which might or might not hold up in an evolutionary simulation. But as the cost/standard of living increases it may be more prone to abrupt failure. And it may be very important to explore this further as a hypothesis in an experimental setting.

The wealth-relative effect remains clear in Figure 11, and seems to be attributable specifically to two sources. TitTat is a poor strategy for the poor if it entails risk. They must take no chances with survival. And as badly as wealthy TitTat does, poor TitTat does much worse. Second, the poor cannot afford to use risky or rarely applicable strategies such as Exploit. In an evolutionary simulation, we might expect these players to be eliminated and replaced by more savvy players (whether intentionally or heuristically savvy). One wouldn't necessarily expect a wealth effect to disappear in an evolutionary simulation, as wealthy players often become poor in simulations, and may find their strategies maladapted.

**Figure 12:**
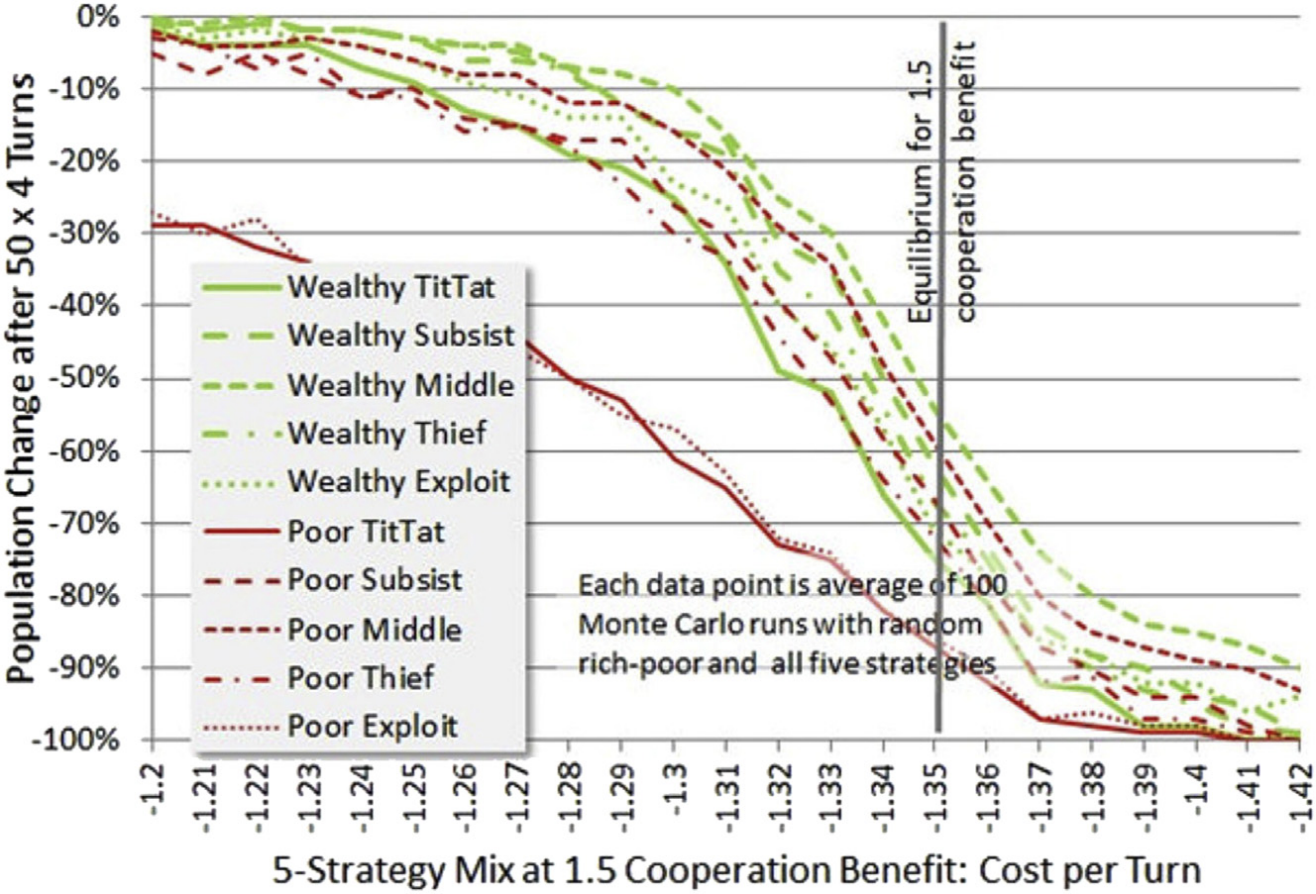
Survival near 1.35 equilibrium for 1.5 cooperation benefit. This figure shows that for a higher cooperation benefit, the die off is less of a “cliff” with respect to small changes in cost per turn, but still heavy. Most of the strategies are closely grouped, but the too badly performing poor strategies are alone at the bottom of performance.

Stewart and Plotkin (2014) report finding that in co-evolution of strategies and payoffs (via Iterated Prisoner's Dilemma) that the benefits of cooperation may be pushed higher, and that tradeoff with the cost of cooperation may lead to a dramatic collapse. A trend toward increases in benefits in cooperation leads via the evidence of Figure 9 to a higher die-off rate at equilibrium, toward which our own arguments have assumed society will be pushed. A high die-off rate (60% over the 50 rounds at a benefit of 1.5, though we do not know exactly what time period this might correspond to) of known relationships is at least not inconsistent with Stewart and Plotkin. At the 1.5 cooperation benefit equilibrium is 1.35 and the population change shown in Figure 12, though not as sharp as at 1.1 benefit, is in heavy die-off.

**Figure 13:**
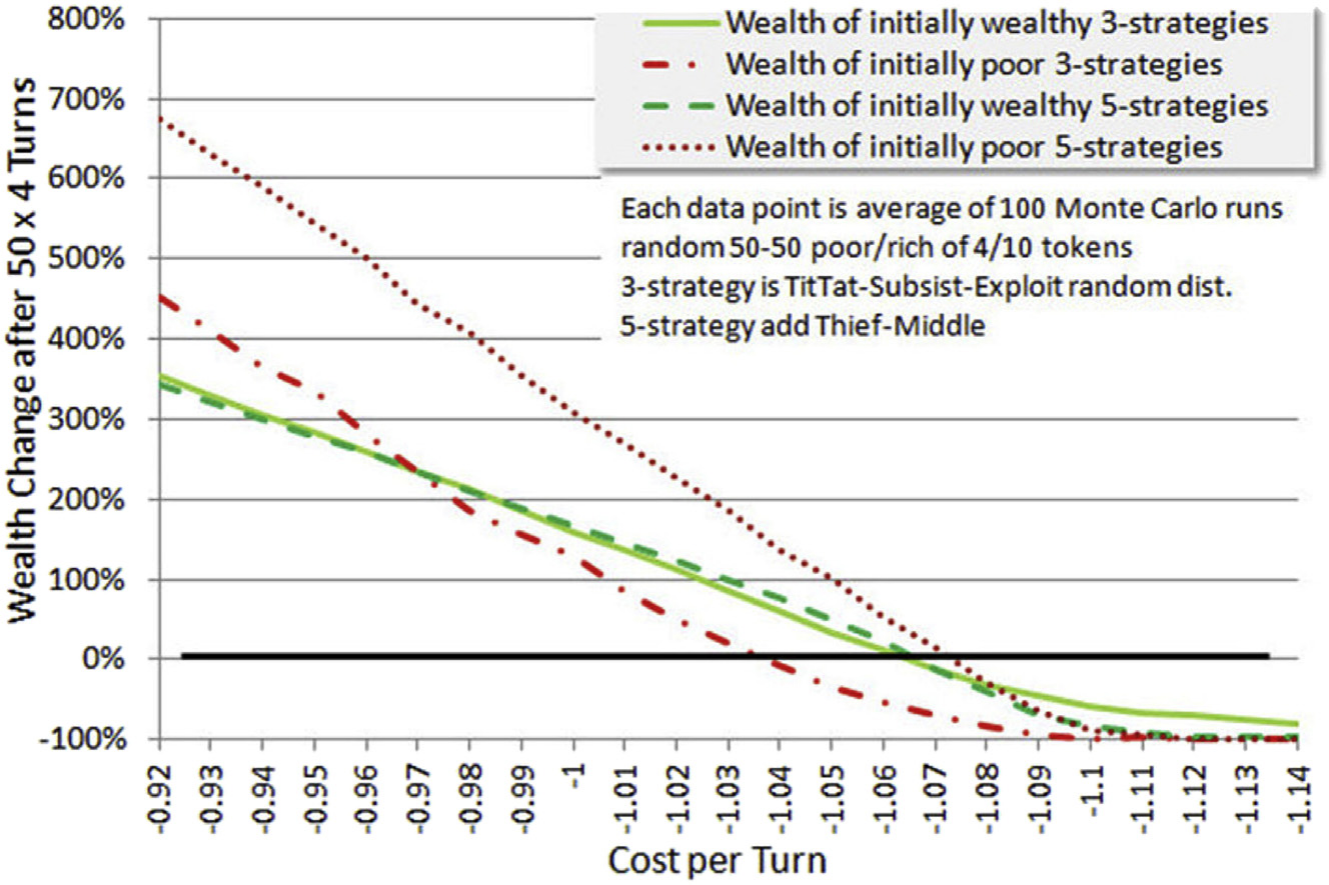
Wealth after 50 rounds by category. Instead of focusing on die off, this chart focuses on total wealth by population segment. The different slopes for rich and poor are attributable to taking percentages off their initial values.

Our final view of the data in Figure 13 shows wealth rather than population. This reveals an “up side” of being poor in our simulated world. They have a small base for measuring future wealth increases, and for any case of cost-per-turn below their equilibrium value the poor get richer faster than the wealthy as a result of this comparative artifact. However, before too much optimism is imbued, note that in the “real world” wealthy “farmers” are not constrained to invest the same 1.07 tokens per turn as everyone else in the game, but may expand their reach in geography and industry to grow wealth exponentially, as ordinarily assumed in finance.

A second reason for presenting Figure 12 is to graphically illustrate just how fast wealth can increase even in the linear fixed-payoff game case, making the poor rich, and moving everyone in a simulation far from the survival threshold. Except for carefully tuned payoffs, everyone either becomes wealthy or dies. This is more than just a reason for lack of note of wealth-relative effects in most simulations. It is importantly a reason why we argue most societies we observe are in fact operating close to equilibrium. Those subsets of society which do exist near equilibrium either die off, or they become wealthy and surrounded by access-controlling staff and isolated by privilege. In either case they become out of the reach of easy study by social scientists.

**Figure 14:**
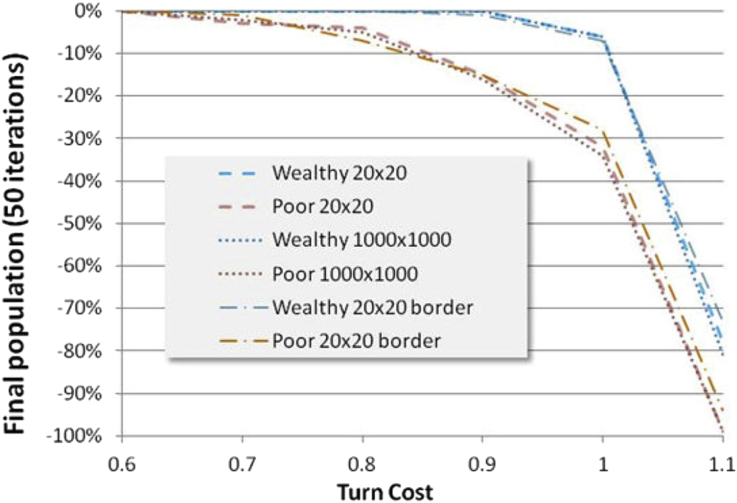
Comparison of 20 × 20 bordered and borderless and 1000 × 1000 borderless networks over a range of turn costs populations for the 3-strategy mix (TitTat, Subsist, Exploit) with initial 50% poor and 50 iterations.

The results presented above were obtained with a 20 by 20 network and initial population of 400. At the border of the network players have fewer transactions, and will benefit if the transactions are negative, or relatively fall behind if the transactions are positive. This was allowed because in the real world such effects exist. Figure 14 shows a comparison over a range of turn costs so the reader can get an idea of the magnitude of this effect. Also shown is a comparison to million-element simulations to demonstrate that there is no significant scaling effect.

**Figure 15:**
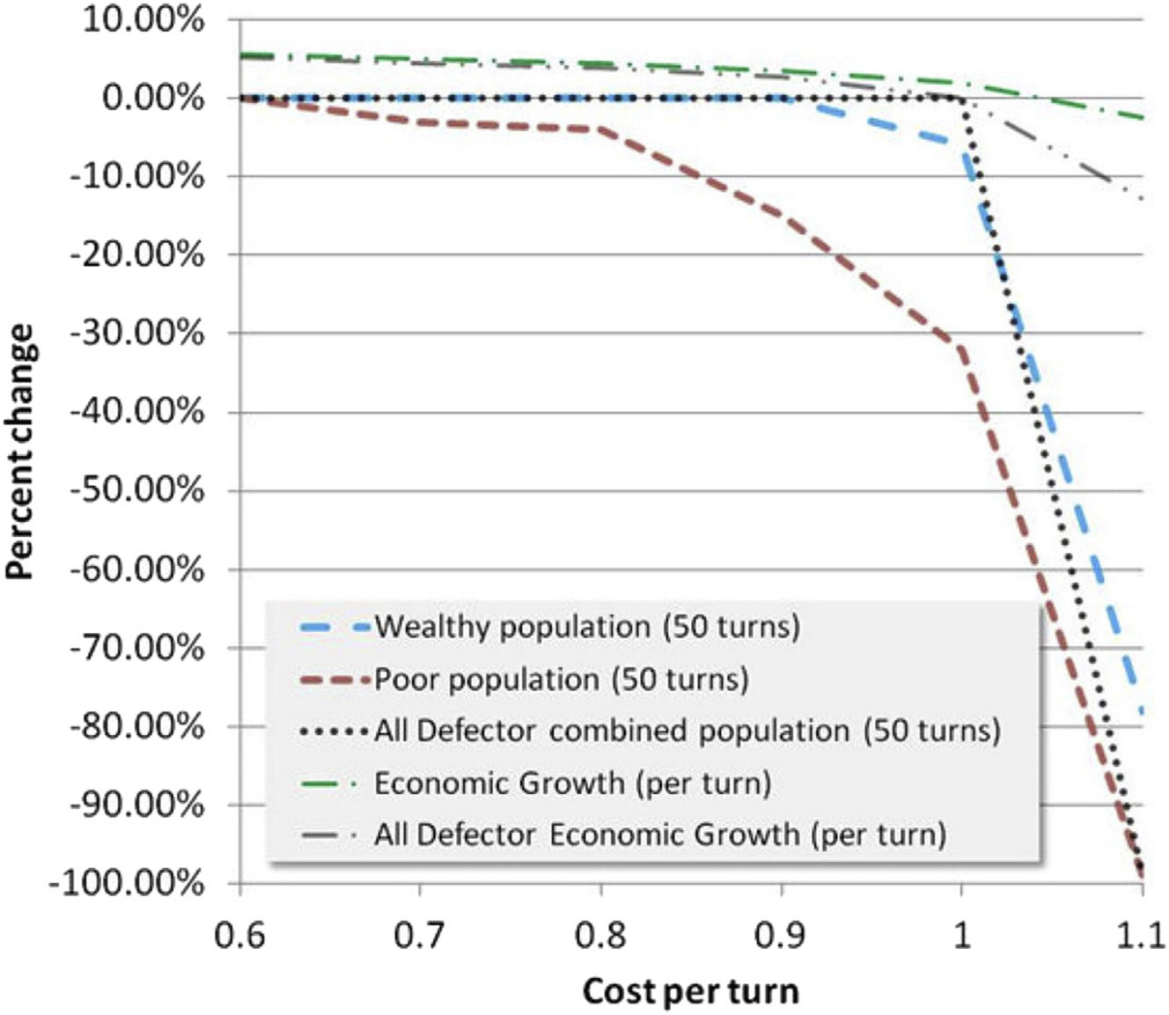
Comparison of cooperating (color legend, 3-strategy mix as in previous figure) and non-cooperating (gray legend, always defect for all players) societies, including population change and average per turn economic growth.

Our next data presentation addresses how the mixed strategy cooperation simulation compares to a completely non-cooperative society, and also presents the total change in wealth over 50 iterations as an average value per iteration. In the Farmer's Game as defined, we could well imagine it to be iterated annually in climates with a single growing season. Our payoff values might be somewhat arbitrary, but operating about equilibrium puts at least the aggregate payoff within reason. The simulation is only in equilibrium for population or wealth, not both at the same time. By showing data over a range of turn costs from no population loss low turn costs to a high turn cost just beyond wealth equilibrium, we provide a general comparison in Figure 15.

The wealth equilibrium turn cost may be taken from where the average economic growth per turn crosses 0%, and as expected it is a bit higher for the cooperating society. Not all of the 10% cooperation payoff is realized in “the economy” due to imperfect strategies, also as expected. About 60–70% of it is realized, which would depend on the strategy mix and is an extensive research topic unto itself. As long as the turn cost is less than the payoff for mutual defection, the non-cooperating society has no population loss (or no bankruptcy if one views this only in financial terms). The individual wealthy net worth to poor net worth for the non-cooperating society changes in predictable ways from 10/4 at the beginning, remaining there if turn cost matches mutual defection payoff, and increasing by the same amount for rich and poor for lower turn costs, making the society more egalitarian.

If an initial cooperating society is of uniform wealth, then regardless of the mix of our five strategies (i.e. excluding unconditional defection) it remains so unless operated beyond equilibrium. And equilibrium increases to a turn cost equal to the increased cooperation payout. Note that this result is probably not reflective of a real society because there are no outside events or calamities in our simulation, no crop failures, no droughts or floods or war, etc. Once inequity is introduced, then the efficiency of cooperation drops.

**Figure 16:**
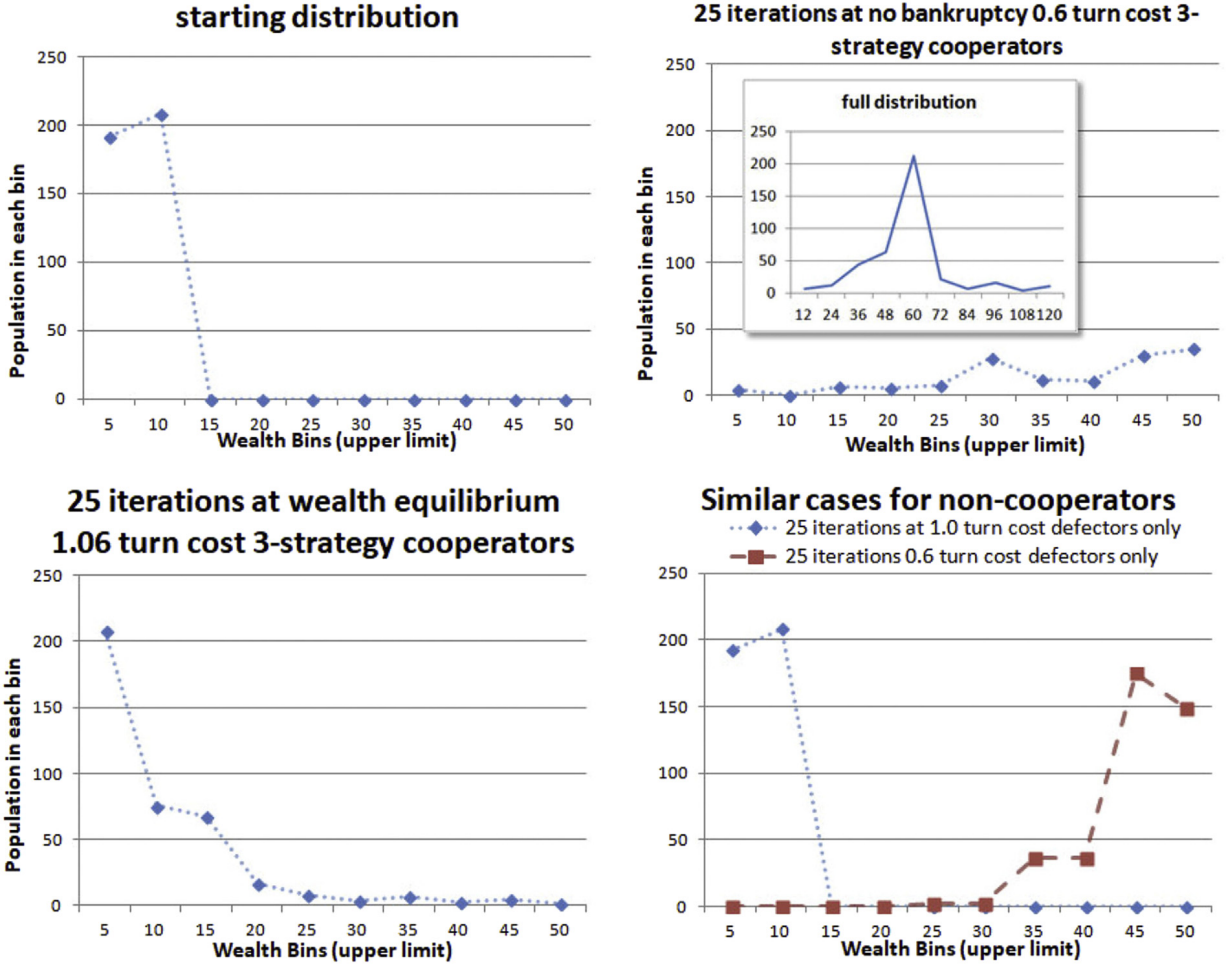
Effects on wealth distribution survival threshold (turn costs of 1.0 or 1.06 vs. 0.6) and cooperation (cooperators at no-die-off 0.6 turn cost).

Figure 16 attempts to show how cooperation and the survival threshold affect future wealth distribution, but approximately in the following way. Survival threshold can essentially be gotten rid of by lowering the cost per turn so that nobody dies. However this creates a disequilibrium in which wealth grows rapidly and is hard to compare. So the number of iterations is varied for each case to keep the range of wealth between 0 and 50 so that histograms can be shown on a common scale. Two cases are shown for the 3-strategy simulation (TitTat, Subsist, Exploit). The first is 20 iterations at the equilibrium cost per turn. While most players persist near their initial wealth, about half of the wealthier participants are spread far up the wealth curve. The second is 8 iterations at 0.6 turn cost, for which nobody dies so there is no effective threshold. The participants remain grouped, but not perfectly, and most slide up in wealth accumulation together.

Two cases are also shown for the non-cooperating always defect society. The first is 25 iterations at 1.0 turn cost, the maximum this society can withstand. Initial wealth distributions remain precisely in place, not changing by a single point. The second is 25 iterations at the 0.6 turn cost, allowing wealth accumulation. Since these simulations were conducted with a border, those on the border have fewer opportunities to accumulate, and fall behind. In the 25 iteration 0.6 turn cost non-cooperative simulation this expands two bins to four as the poor and wealthy on the border accumulate less. However, in 0.6 turn cost cooperative simulation, the two bins spread to 5 after only 8 iterations, indicating some dispersion of wealth by cooperation, as there is effectively no threshold at this turn cost. Such dispersion obviously depends on the cooperation strategies, and might be a topic for further investigation.

One cannot help but notice when reviewing Figures 15 and 16 that for the game parameters used, the cooperative society has an advantage in total wealth accumulation, but is decidedly inferior in the cost per turn it can withstand without population loss (or personal financial failure, if it is interpreted as a financial model). This might suggest that a much higher cooperation benefit than 10% is desirable in a cooperating society, otherwise non-cooperation is more predictable and stable. A higher cooperation benefit also has an influence on inducement to cooperation, but some people remain persistently erratic cooperators as reported by Capraro et al. (2014). In the simulation data, Figures 9 amd 12 provide results for higher cooperation benefit (up to 50%), indicating a sharper decline near equilibrium for higher cooperation benefit. However, the decline rate varies greatly with strategy, and the non-cooperating society simulation has quite a sharp cutoff. If feedback is present in society a sharp decline would tend to lead to instability, and the presence of poor individual strategies with gentler decline slopes might be advantageous to society.

In a human evolutionary setting, the cooperation benefit for something like agriculture is quite complex and changes over time. At first it is perhaps a modest gain. As cultivators take over more and more land and institute concepts like land ownership, non-participation can become less viable. Both growing crops and food stockpiles attract theft or attack and require defense. When this condition occurs, non-participation acquires a further vulnerability. The accumulated effect is so great that only a few members of modern civilization choose to live alone or in small groups as hunter gatherers.

The dispersal of wealth may actually promote the evolution of cooperation. Perc and Szolnoki (2008) report that a power law distribution of fitness (aka wealth) is optimal. Near equilibrium simulations such as the 1.06 turn cost cooperators in Figure 16 indeed produced a distribution qualitatively similar to a power law. This bears further investigation, but could be a significant factor in the evolution of cooperation. Evolution does not necessarily produce the most fair or egalitarian societies. It maximizes fitness, or survivability of the individuals. If a large number of groups are available to support group evolution (Maynard Smith, 1976) then it maximizes group survivability. The presence of a large labor force and a number of extremely fit (wealthy) people might increase the options a society has under some circumstances. Under some conditions, the labor block might prevail. In others, the high fitness individuals might prevail over the opposing conditions. A power law preserves these two extremes and provides some in between.

## 6 Discussion

### 6.1 Relation to experimental data

The experiment conducted by Buchan et al. is extremely important. The results of our paper do not contradict their findings, which are empirical, or their reasoning that these findings are likely due to globalization. However, there is more than one way in which they could depend on globalization. They could be due to the different connectivity in the globalized countries, or due to the greater affluence of those countries which may well be in part due to globalization, in which case all citizens in the participating countries need to share in the benefits or the level of cooperation may not be maintained.

Other areas of social change exhibit the same kind of ambiguity between social network and affluence as cause or effect. Sander and Putnam (2010) point out that in the early 1960s more than half of all Americans said they trusted others, but it was only one third in 2010. Since 2001 only the upper-middle-class young people have remained civically engaged while pay-for-play extracurricular activities and teaching-to-the-test have encouraged others to drop out, according to Sander and Putnam. Can this be corrected by encouraging adults and youth to be more socially connected? Or do we have to address the economic mechanisms by which the affluence gap has widened as Putnam et al. (2012) seem to suggest two years later?

The amount participants were willing to risk in the Buchan et al. study is only a fraction of a day's pay ranging from a tenth to a half, small when compared to the investment necessary to address real public goods issues such as climate change. Risk was an important element in our framing of the game to decide how to apply relative payoff value. This will vary with both game structure and knowledge the participants have about each other, in this case very little. Apparently it also varies with network structure, since the randomized (de-clustered) network better fit the Buchan et al. data. Are low clustering and stranger interaction both proxies for some kind of risk? Depending on simulation model parameters there likely are different effects, for example clusters of cooperators being “food” for defectors in basic scenarios. In memory models where Tit-for-Tat has evolved, there may be greater risk of retaliation from strangers. Either of these could be dependent on the magnitude of the temptation/defection, and explain the crossover in Figure 1 at high temptation where the randomized network becomes the higher cooperating one.

With Lozano et al. partly confirmed by reconciliation with Buchan et al.‘s experimental data, the principle of relativity of temptation/payoff values introduced, and a possible relation between strangeness and network structure at the hypothesis stage, many interesting possibilities for investigation are apparent. We can postulate a model of a class-structured society in which individual cooperation depends upon the magnitude of the payoffs of each “game.” Would such a model show classes cooperating with their peers, but taking risk-averse strategies (whether they be cooperation or defection) when cooperating with more wealthy classes and vice versa? If in such a model one friend or relative becomes wealthier than another, would the dry mathematical model produce a change in cooperative behavior we might call “envy”? Do class structures play a role in evolutionary stability?

### 6.2 Simulation findings and relation to political economy

The development of the wealth-relative hypothesis and re-interpretation of experimental data focused on the decision processes of individuals relative to their wealth, history, and threshold for survival. The simulation results obtained from this assumption, as summarized in Figure 16, appear to show effects on the distribution of wealth, in other words political economy. As already mentioned this poses interesting questions on the self-reinforcement of cooperation in an evolutionary setting.

Cooperation and its development are studied by sociologists, physicists, economists, mathematicians, evolutionary biologists, computer scientists and others. Each may think of it in their own terminology. In this section we consider the terminology of economics. The initial gift of fitness to promote hoped-for cooperation appears similar to a risky investment. There is no guarantee of return. But empirically it is found that excess returns accrue if conditions are favorable to cooperation. Cooperation in the presence of methods of punishment or coercion appears similar to investing in bonds or other debt instruments where an explicit and enforceable promise of returns is made. Equalization among different types of returns should occur. Somewhat to the theoretical puzzlement of economists, the unguaranteed “equity” returns are substantially higher than the enforced or “debt” returns. This difference is called the Equity Premium (Mehra, ed., 2008). It would seem that models of cooperation should only be pronounced “accurate” if they too produce this premium. Neither the model herein nor any model we've seen rises to this level, so challenges remain.

**Figure 17:**
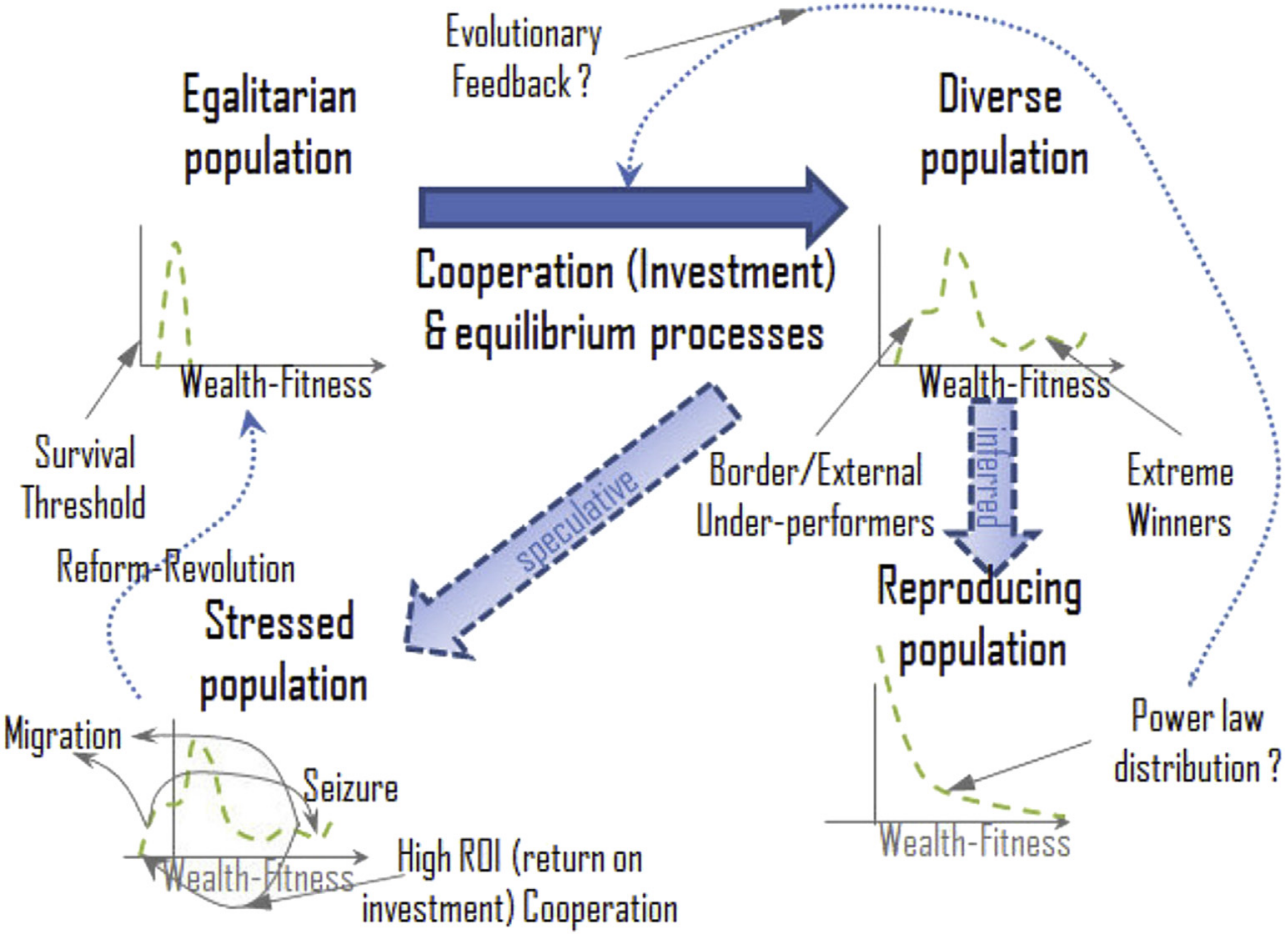
Summary of simulation findings in terms of wealth redistribution (top section) and suggestions for related research (bottom section, see text).

We could summarize the simulation result graphically as depicted in the top portion of Figure 17, the transition indicated by the solid blue arrow. An initial society with one or two wealth levels will find the wealth redistributed asymmetrically by the probabilistic nature of cooperation results, differing opportunities for cooperation (as for example in the simulation from boundary effects), and a survival threshold under near equilibrium (primarily responsible for the asymmetry).

Other parts of the figure represent inferred (downward transition on the right) or speculative (diagonal transition on the left) transitions which are suggested as starting hypotheses for further investigation. The downward transition is inferred by assuming a population in which the wealth status of children is related to the wealth status of parents. In the political economy interpretation, the game payoff and wealth accumulation is *not* fitness, it is only wealth. A separate relation is needed to arrive at consequential fitness. Birth rate may be negatively correlated with wealth as is the case in many populations of interest (Vandenbroucke, 2016). In Figure 16 the simulation at a near equilibrium turn cost of 1.06 produces a wealth distribution with larger populations at lower wealth levels. A negative wealth to birth rate correlation will presumably exaggerate such a distribution.

The transition back to the left in Figure 17 speculates about a stress on the society which brings the survival threshold well into the populated region. The response of the society may depend on many things, and only a few possible responses are indicated. Need-based sharing may prompt individuals to make potentially high return social investments in those whose survival is threatened (Smith et al., 2019). A simple example is disaster relief. People may cooperate more with their neighbors also suffering from the disaster, as well as receiving aid from segments of the population not affected. But under conditions less favorable to cooperation (about which we do not speculate here) members of the population may flee (migration) or engage in conflict (seizure of assets, such as looting), or may attempt redistribution of wealth by reform or revolution rather than voluntary or market actions. These decisions then may affect future potential for cooperation.

## 7 Conclusion

There are three essential messages resulting from this investigation.

Cooperating involves risk taking. Whether the risk is in cooperation or defection depends on the payoff matrix. When survival is at stake, rational players will consider risk over cooperation. The amount of risk will be perceived through the lens of relative wealth. This hypothesis successfully explains several complex results from real life experiments and was also found in simulation under near equilibrium with a survival threshold.

When cooperation is the higher risk option as in variations of Prisoner's Dilemma, such as the Farmer's Game introduced here, it may not benefit those near the survival threshold, which we term “the poor” in this investigation. More generally, one should be cautious in promoting by persuasion or policy life and financial strategies to those near the survival threshold which work for those far from the threshold. Some individuals such as the transient homeless might be employing a strategy for “skipping” turn cost until resources are available for a recovery, a sort of analogy to endospores. This is similar to the high performing “poor subsist” strategy, but even more extreme. In general strategies which became risk adverse near the threshold did better in our simulations, with “Middle” performing the best for both poor and wealthy participants by avoiding risk within two full iterations (8 transactions) of the threshold.

Cooperation involves uncertainty, and increases the dispersion of wealth. Separately a survival threshold has this effect, and also produces asymmetry in the distribution. Non-cooperation is relatively more predictable and conservative of wealth structure. Boundary conditions and external events produce wealth inhomogeneity, and cooperation with a survival threshold spreads it further. Non-cooperation with a surplus can, under equitable payoff conditions, lead to lower wealth dispersion by increasing everyone's wealth, though this is a non-equilibrium condition. It seems then that at least some of the social phenomena commonly attributed to economic and political ideologies are produced by cooperation. To the extent cooperation, by virtue of capturing higher payoffs, provides for greater total society wealth accumulation, this might suggest societies with more equitable wealth distribution have an intrinsic tendency to lower total wealth. To the extent cooperation produces wealth dispersion approximating the dispersion needed to promote cooperation provides an evolutionary motive force independent of and in some cases at odds with notions of social justice. These preliminary observations need to be investigated further.

The hope embodied in this paper is that sufficient introduction of the topic has been made that some researchers will consider it, ever mindful of the utility of attempting to reconcile theoretical and simulation results with empirical experiment, which is also facilitated by the wealth-relative formulation. Cooperation theory has made rapid advances since the early 1980s, and may now be able to be applied in the real world. If appropriately scaled and calibrated it may help safely and prudently “engineer our society” as Ezaki et al. (2016) suggest. But as cooperation is very valuable, it is also subject to the misadventures of hasty pursuit.

## Data availability statement

The reader may find an interactive version of the simulation at http://mc1soft.com/papers/wealthrelative/ and also uploaded as supplementary data to this manuscript, along with all data from this paper in spreadsheet format.

## Declarations

### Author contribution statement

Robert Shuler: Conceived and designed the experiments; Performed the experiments; Analyzed and interpreted the data; Contributed reagents, materials, analysis tools or data; Wrote the paper.

### Funding statement

This research did not receive any specific grant from funding agencies in the public, commercial, or not-for-profit sectors.

### Competing interest statement

The author declares there are no competing interests. This work does not necessarily represent the opinions of the National Aeronautics and Space Administration.

### Additional information

Supplementary content related to this article has been published online at https://doi.org/10.1016/j.heliyon.2019.e02958.

## Appendix A Supplementary data

simulation_code simulation_code

simulation_data simulation_data
