# Supplementary material for: Wealth-relative effects in cooperation games
Source: Heliyon. 2019 Dec 3;5(12):e02958. doi: 10.1016/j.heliyon.2019.e02958 (PMC6909069; doi:10.1016/j.heliyon.2019.e02958)
Supplement: simulation_code [file mmc1.zip › hly_2958_simulation_code_mmc1.html]

Wealth-relative Payoff in Cooperation Games Simulation 


Wealth-relative Payoff in Cooperation Games - Simulation ver. 2
  
© Robert Shuler 2018, contact
This simulation uses a version of Prisoner's Dilemma which I call the FARMER's GAME (or one could call it "Survivor";).
You can change the payoff matrix (as long as it is symmetric) to explore other games.
My object was to explore several wealth-relative effects suggested in a paper by
Ito et. al. which are not normally considered
(i.e. standard game theory assumes the absolute values of payoffs make no difference, and if evolutionary,
replacement criterion is copying a neighboring strategy, not identifiable death of a player). The sim is only evolutionary
in the restricted sense that wealth accumulates and players may die. Strategies do not evolve (except that they may depend
on wealth) and are not copied, and new players are not created. Full evolutionary sims would take too long in Javascript,
and by evolving strategies and creating players would actually obscure measures of particular wealth-strategy combinations.
However, this sim allows questions about survival thresholds and the relative merit of defined strategies to be quickly answered.
See below for copy/mod permissions.  


**Game Cost Per Turn (interaction):** cost (negative) or stipend (positive)  

**Note:** If player wealth drops to or below zero, they are removed from game (see mortality action)  

**Payoff matrix:** (symmetric, player on left payoff, other player move on top)  
            C                   D  
C   
       
D   
    

**Strategy Mix:** (initial distribution is random, equal weighted)  
 *B - Wealth Blind (base):* Tit for tat with
% forgiveness  
 *S - Subsistence:* If I may not survive then Defect, else Tit for Tat   
 *M - Middle Class:* If I may not survive
 turns then Defect, else Tit for Tat   
 *T - Thief:* If other is
 times as wealthy then Defect, else Tit for Tat  
 *E - Exploiter:* If I am
 times as wealthy then Defect, else Tit for Tat  
 *D - Defect Always*  

**Wealth Distribution:**   
Density of poor (P)
% at
 game tokens  
All remaining are wealthy (W) at
 game tokens  

**Network type:** currently fixed at LATTICE. **Size:**
 by
  
 **Borderless** (wrap rows and columns)
 **Print initial lattice?** (in results tab)
 **Print evolving lattice?** (requires clicks, not for high iteration count;
drag tab to separate window to actually see it)
**Mortality action:** currently fixed at CONNECT NEXT NEIGHBOR  
**Number of Iterations:**
 (each iteration is one interaction with all four neighbors)  
**Output every:**
 iterations   


 
  
  
or  sim, iterate for
 runs  
  
**Note:** After pressing start, it may take a while depending on number of iterations. Results will open in new tab.  
**Note:** Two letter column headings denote wealth category and strategy, e.g. WE, PS, etc.  
  
  
 max bin value (use 0 for automatic, there will be
 bins)  

**Privacy:** All computation is done on your computer. No data is transmitted over the internet
or stored on the server.  

**Copy and Modification Permissions:** You may copy and modify the sim if you give credit to the source.
(In Firefox: *Tools->Web Developer->Page Source*)
The simplest way to try new strategies is to usurp one of my strategy letters and change the function GAME. The data stored
for each player is defined only once by the assignment 'loc[i]=...' in INITIALIZE LATTICE. You can add strategy parameters there,
or links for other network topologies.
